# Overexpression of protein kinase STK25 in mice exacerbates ectopic lipid accumulation, mitochondrial dysfunction and insulin resistance in skeletal muscle

**DOI:** 10.1007/s00125-016-4171-5

**Published:** 2016-12-16

**Authors:** Urszula Chursa, Esther Nuñez-Durán, Emmelie Cansby, Manoj Amrutkar, Silva Sütt, Marcus Ståhlman, Britt-Marie Olsson, Jan Borén, Maria E. Johansson, Fredrik Bäckhed, Bengt R. Johansson, Carina Sihlbom, Margit Mahlapuu

**Affiliations:** 1 Lundberg Laboratory for Diabetes Research, Department of Molecular and Clinical Medicine, University of Gothenburg, Sahlgrenska University Hospital, Blå stråket 5, SE-41345 Gothenburg, Sweden; 2 Wallenberg Laboratory, Department of Molecular and Clinical Medicine, University of Gothenburg, Sahlgrenska University Hospital, Gothenburg, Sweden; 3 Proteomics Core Facility, University of Gothenburg, Gothenburg, Sweden; 4 Department of Physiology, Institute of Neuroscience and Physiology, University of Gothenburg, Gothenburg, Sweden; 5 Novo Nordisk Foundation Center for Basic Metabolic Research, University of Copenhagen, Copenhagen, Denmark; 6 Institute of Biomedicine, Electron Microscopy Unit, University of Gothenburg, Gothenburg, Sweden

**Keywords:** Ectopic lipid storage, Insulin resistance, Mitochondrial dysfunction, Skeletal muscle

## Abstract

**Aims/hypothesis:**

Understanding the molecular networks controlling ectopic lipid deposition and insulin responsiveness in skeletal muscle is essential for developing new strategies to treat type 2 diabetes. We recently identified serine/threonine protein kinase 25 (STK25) as a critical regulator of liver steatosis, hepatic lipid metabolism and whole body glucose and insulin homeostasis. Here, we assessed the role of STK25 in control of ectopic fat storage and insulin responsiveness in skeletal muscle.

**Methods:**

Skeletal muscle morphology was studied by histological examination, exercise performance and insulin sensitivity were assessed by treadmill running and euglycaemic–hyperinsulinaemic clamp, respectively, and muscle lipid metabolism was analysed by ex vivo assays in *Stk25* transgenic and wild-type mice fed a high-fat diet. Lipid accumulation and mitochondrial function were also studied in rodent myoblasts overexpressing STK25. Global quantitative phosphoproteomics was performed in skeletal muscle of *Stk25* transgenic and wild-type mice fed a high-fat diet to identify potential downstream mediators of STK25 action.

**Results:**

We found that overexpression of STK25 in transgenic mice fed a high-fat diet increases intramyocellular lipid accumulation, impairs skeletal muscle mitochondrial function and sarcomeric ultrastructure, and induces perimysial and endomysial fibrosis, thereby reducing endurance exercise capacity and muscle insulin sensitivity. Furthermore, we observed enhanced lipid accumulation and impaired mitochondrial function in rodent myoblasts overexpressing STK25, demonstrating an autonomous action for STK25 within cells. Global phosphoproteomic analysis revealed alterations in the total abundance and phosphorylation status of different target proteins located predominantly to mitochondria and sarcomeric contractile elements in *Stk25* transgenic vs wild-type muscle, respectively, providing a possible molecular mechanism for the observed phenotype.

**Conclusions/interpretation:**

STK25 emerges as a new regulator of the complex interplay between lipid storage, mitochondrial energetics and insulin action in skeletal muscle, highlighting the potential of STK25 antagonists for type 2 diabetes treatment.

**Electronic supplementary material:**

The online version of this article (doi:10.1007/s00125-016-4171-5) contains peer-reviewed but unedited supplementary material, which is available to authorised users.

## Introduction

Type 2 diabetes is strongly associated with ectopic lipid deposition within non-adipose tissue, which actively contributes to the development of insulin resistance [1–3]. Skeletal muscle plays an important role in the pathophysiology of type 2 diabetes accounting for more than 70% of whole body glucose use [4]. Thus, approaches that can suppress ectopic lipid deposition within the skeletal muscle, and increase the responsiveness of muscle to insulin, offer a potential for the development of new therapies for diabetes.

In the search for novel targets that contribute to the pathogenesis of insulin resistance and type 2 diabetes, we recently described serine/threonine protein kinase 25 (STK25; also referred to as YSK1 or SOK1), a member of the sterile 20 (STE20) kinase superfamily [5], as a central regulator of ectopic lipid accumulation, and whole body glucose and insulin homeostasis [6–11]. STK25 is broadly expressed in mouse, rat and human tissues [10–13]. Previous studies have shown that STK25, present in the Golgi complex, regulates cell polarisation and migration in different cell types [14–17]. It is also reported that in cells subjected to extreme stresses, STK25 enters the nucleus and induces cell death [18, 19]. We found that in mice fed on a high-fat diet, transgenic mice overexpressing STK25 display hyperinsulinaemia and impaired whole body glucose and insulin homeostasis compared with wild-type littermates [10]. Reciprocally, our studies showed that, compared with wild-type littermates, *Stk25* knockout mice are protected against systemic glucose intolerance and insulin resistance induced by a high-fat diet [6]. Notably, we found that in both mouse and human liver cells, STK25 is localised on the surface of cytosolic lipid droplets [7, 8]. We observed that increased STK25 abundance in mouse liver and human hepatocytes enhances fat deposition in intrahepatocellular lipid droplets by suppressing lipolytic activity and thereby fatty acid release for β-oxidation and triacylglycerol secretion; the reciprocal effect was seen with STK25 knockdown [7, 8]. Furthermore, we found a significant positive correlation between *STK25* mRNA expression and fat content in human liver biopsies [8, 9]. Moreover, *STK25* mRNA levels were higher in the skeletal muscle of individuals with type 2 diabetes than in healthy volunteers [11].

On the basis of our previous findings, which reveal a central role of STK25 in control of hepatic fat deposition and systemic insulin sensitivity [6–10], we hypothesised that STK25 is also involved in regulation of ectopic lipid storage and insulin responsiveness in skeletal muscle. Here, we provide the first evidence to support the key cell-specific role of STK25 in the excessive accumulation of intramyocellular lipids in the context of chronic exposure to dietary lipids, which is associated with suppressed mitochondrial function, reduced endurance exercise capacity and exacerbated insulin resistance in skeletal muscle.

## Methods

### Animals

The generation of *Stk25* transgenic mice, where mouse *Stk25* expression in the targeting construct is controlled by chicken β-actin promoter, and the subsequent breeding with C57BL/6NCrl mice (Charles River, Sulzfeld, Germany), have been described previously [10]. From the age of 6 weeks, male transgenic mice and wild-type littermates were fed a pelleted high-fat diet (45% kilocalories from fat; D12451; Research Diets, New Brunswick, NJ, USA). At the age of 24 weeks, the mice were killed after 4 h of food withdrawal. Gastrocnemius skeletal muscle samples were collected for histological analysis (see Histology and immunofluorescence) or snap frozen in liquid nitrogen and stored at −80°C for analysis of protein and gene expression (see ESM Fig. 1 for a schematic overview). All animal experiments were performed after approval from the local Ethics Committee for Animal Studies at the Administrative Court of Appeals in Gothenburg, Sweden, and followed appropriate guidelines.

### Histology and immunofluorescence

Gastrocnemius muscle samples were embedded in optimal cutting temperature mounting medium (Histolab Products, Gothenburg, Sweden) and frozen in liquid nitrogen followed by cryosectioning and staining with haematoxylin-eosin (H-E; Histolab Products), Nile Red (Sigma-Aldrich, St Louis, MO, USA) or MitoTracker Red (Thermo Fisher Scientific, Waltham, MA, USA). Enzymatic stainings were performed as previously described [20]. For immunofluorescence, sections were incubated with primary antibodies followed by incubation with secondary antibodies (see ESM Table 1). Gastrocnemius muscle samples were also fixed with 4% formaldehyde in phosphate buffer (Histolab Products), embedded in paraffin, sectioned and stained with Picrosirius Red (Histolab Products) or Periodic acid–Schiff (PAS; Sigma-Aldrich). Ultrastructural analysis of gastrocnemius muscle was performed by transmission electron microscopy (TEM; LEO 912AB; Omega; Carl Zeiss NTS, Oberkochen, Germany) as previously described [21]. Gastrocnemius muscle homogenates were analysed using a Hydroxyproline Colorimetric Assay Kit (Sigma-Aldrich) and a Triglyceride Calorimetric Assay Kit (Biovision, Mountain View, CA, USA).

### Cell culture and transient overexpression

L6 myoblasts (*Rattus norvegicus*, American Type Culture Collection, Manassas, VA, USA) were maintained as described [11] and transfected with pFLAG-*Stk25* (cytomegalovirus promoter; GeneCopoeia, Rockville, MD, USA) or an empty control plasmid using Lipofectamine 2000 (Invitrogen, San Diego, CA, USA). Cells were incubated with 50 μmol/l oleic acid for 24 h and stained with Oil Red O or MitoTracker Red as described [8]. Palmitate oxidation was measured as previously described [11]. Cells have been demonstrated to be free of mycoplasma infection by use of the MycoAlert Mycoplasma Detection kit (Lonza, Basel, Switzerland).

### Western blot and quantitative real-time PCR

Western blotting was performed as previously described [7] in gastrocnemius muscle of *Stk25* transgenic and wild-type mice and/or transfected L6 myoblasts using anti-STK25, anti-adipose triacylglycerol lipase (ATGL), anti-hormone-sensitive lipase (HSL), anti-PLIN2 and anti-actin primary antibodies (see ESM Table 1). The anti-STK25 antibody has been validated by using *Stk25*-knockout mice [6]. Quantitative real-time PCR was performed in gastrocnemius muscle of *Stk25* transgenic and wild-type mice and transfected L6 myoblasts using the ABI Prism 7900HT Sequence Detection System (Applied Biosystems, Foster City, CA, USA) as described [10] (see ESM Table 2).

### Ex vivo measurement of lipid metabolism

The oxidation rate of palmitate was measured in quadriceps muscle homogenates as described previously [7]. Oleic acid uptake and triacylglycerol synthesis from [^14^C]-oleic acid were measured in isolated extensor digitorum longus (EDL) and soleus muscles as described [22, 23].

### In vivo assessment of exercise performance and insulin sensitivity

Endurance exercise was assessed by treadmill running until the mice reached fatigue (see ESM Methods). Insulin sensitivity was measured by euglycaemic–hyperinsulinaemic clamp as previously described [10] using an insulin infusion rate of 7 mU/min/kg (see ESM Methods).

### Liquid chromatography mass spectrometry analysis

Gastrocnemius muscle samples were heat stabilised and prepared, including tryptic digestion, chemical labelling for relative quantification, enrichment of phosphopeptides and pre-fractionation, as described in ESM Methods. Liquid chromatography mass spectrometry (LC-MS)/MS of these combined tandem mass tagged labelled samples was performed on an Orbitrap Fusion Tribrid MS interfaced to an Easy-nLC 1000 (Thermo Fisher Scientific).

### Statistical analysis

Statistical significance between groups was calculated with an unpaired two-tailed Student’s *t* test or by two-way ANOVA followed by Tukey post hoc test. A *p* < 0.05 was considered statistically significant.

## Results

### Evidence for muscle damage in *Stk25* transgenic mice, with fibre type composition remaining unaltered

We previously showed that STK25 is highly expressed in mouse, rat and human skeletal muscle [11]. Here we found that endogenous STK25 protein was 2.1 ± 0.3-fold higher in white portions of the gastrocnemius muscle (predominantly type IIb fibres) compared with red portions (predominantly type IIa fibres) in wild-type mice (ESM Fig. 2a). Nonetheless, the endogenous STK25 protein was detected by immunofluorescence in all fibre types (ESM Fig. 2b). STK25 was markedly increased in both red and white portions of *Stk25* transgenic vs wild-type muscle (ESM Fig. 2a, b).

**Fig. 1:**
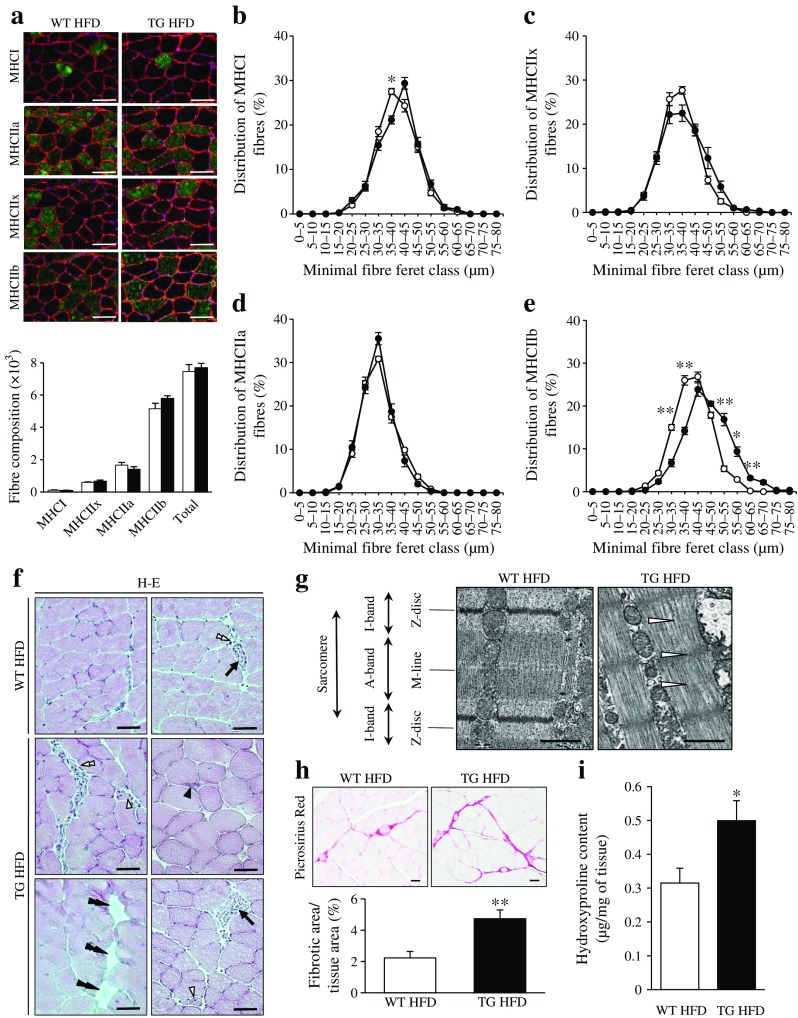
Morphology and fibre composition in gastrocnemius muscle of *Stk25* transgenic and wild-type mice. (**a**) Representative immunofluorescence images double-stained with antibodies for MHC type I, IIa, IIx or IIb (green) and laminin (red); nuclei stained with DAPI (blue). White bars, wild-type mice fed high-fat diet; black bars, transgenic mice fed high-fat diet. Scale bar, 50 μm. Histogram shows quantification of fibre types (**b**–**e**) Fibre size distribution. White circles, wild-type mice fed high-fat diet; black circles, transgenic mice fed high-fat diet. (**f**) Representative images stained with H-E showing the presence of intracellular inclusions (black single arrowhead), small angular degenerating fibres (open single arrowheads), focal necrosis (arrows), infiltration of mononuclear inflammatory cells (open double arrowheads) and adipocyte replacement (black double arrowheads). Scale bar, 50 μm. (**g**) Representative electron micrographs from longitudinal sections showing disrupted sarcomere organisation (open arrowhead). Scale bar, 1 μm. (**h**) Representative images stained with Picrosirius Red (scale bar, 50 μm) and quantification of Picrosirius Red staining. (**i**) Hydroxyproline content in the muscle extract. In (**a**–**e**, **h**–**i**) data are mean ± SEM from 5–8 mice per genotype. *p < 0.05; **p < 0.01. HFD, high-fat diet; TG, transgenic; WT, wild-type

Fibre type proportions, as identified by myosin heavy chain (MHC) immunochemistry, were unchanged in *Stk25* transgenic compared with wild-type muscle (Fig. 1a); type IIb fibres of transgenic muscle appeared slightly hypertrophic, while no shift in diameter was found in any other fibre type (Fig. 1b–e). Examination of H-E-stained sections revealed evidence of muscle damage in *Stk25* transgenic mice as indicated by intracellular inclusions, small angular degenerating fibres, focal necrosis, infiltration of mononuclear inflammatory cells and adipocyte replacement; these features were rarely seen in wild-type muscle (Fig. 1f). TEM showed the presence of well-defined myofibrils and sarcomeric pattern with organised A- and I-bands, Z-discs and M-lines in wild-type muscle (Fig. 1g). In contrast, *Stk25* transgenic muscle fibres displayed disorganised myofibril architecture and irregularities of sarcomere elements (Fig. 1g).

Picrosirius Red staining for collagen revealed that perimysial fibrosis was increased in *Stk25* transgenic vs wild-type muscle and endomysial fibrosis, not present in wild-type muscle, was readily observed in transgenic muscle (Fig. 1h). Consistently, hydroxylated proline, a main constituent of collagens, was 1.6 ± 0.2-fold higher in *Stk25* transgenic muscle homogenates (Fig. 1i).

### STK25 overexpression in mice augments fat storage and impairs mitochondrial function in skeletal muscle

**Fig. 2:**
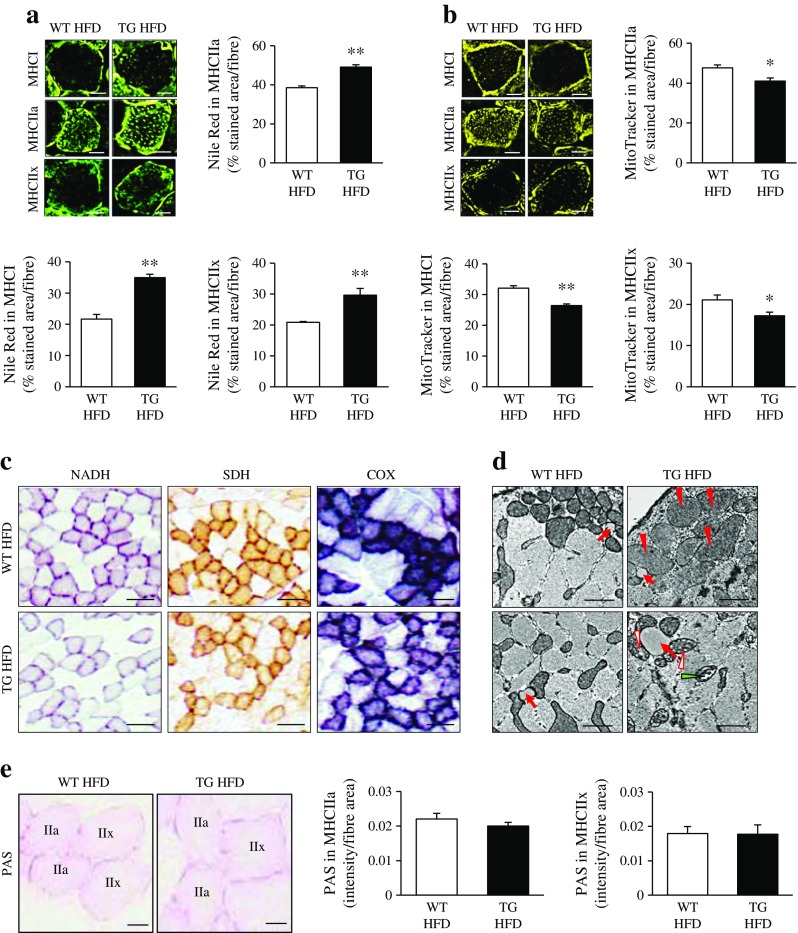
Lipid storage, mitochondrial function and glycogen content in gastrocnemius muscle of *Stk25* transgenic and wild-type mice. (**a**) Representative immunofluorescence images stained with lipophilic Nile Red dye (green). Scale bar, 15 μm. Histograms show quantification of Nile Red staining. (**b**) Representative immunofluorescence images stained with MitoTracker Red (yellow). Scale bar, 15 μm. Histograms show quantification of MitoTracker staining. (**c**) Representative histochemical staining for NADH, SDH and COX activities. Scale bar, 50 μm. (**d**) Representative electron micrographs from cross-sections showing lipid droplets (red arrows) and mitochondria, which are swollen (red arrowheads), display disarrayed cristae and reduced electron density of the matrix (open arrowheads), and internal vesicles (green arrowhead). Scale bar, 2 μm. (**e**) Representative images stained with PAS (scale bar, 15 μm) and quantification of PAS staining. In (**a**, **b** and **e**) data are mean ± SEM from six mice per genotype. *p < 0.05; **p < 0.01. HFD, high-fat diet; TG, transgenic; WT, wild-type

We measured the intramyocellular lipid accumulation in the fibre types with the highest lipid content (type I, IIa and IIx) in gastrocnemius muscle of *Stk25* transgenic and wild-type mice fed a high-fat diet. The relative area of these muscle fibres staining with lipophilic dye Nile Red was approximately 1.3- to 1.6-fold higher in *Stk25* transgenic mice (Fig. 2a). In contrast, the relative area of these muscle fibres staining with MitoTracker Red, a fluorescent dye that specifically accumulates within respiring mitochondria, was approximately 1.2-fold lower in *Stk25* transgenic mice compared with wild-type mice (Fig. 2b). Consistent with a reduced MitoTracker Red signal, histochemical stainings revealed that *Stk25* transgenic muscle exhibited repressed pigment retention in enzymatic activity assays for NADH, succinate dehydrogenase (SDH) and cytochrome c oxidase (COX), commonly used as markers of oxidative metabolism (Fig. 2c).

TEM demonstrated that a significant fraction of mitochondria in *Stk25* transgenic, but not wild-type, muscle were structurally distorted and appeared swollen, and displayed disarrayed cristae, reduced electron density of the matrix and/or internal vesicles (Fig. 2d, ESM Fig. 3). TEM also revealed the presence of large lipid droplets in transgenic but not wild-type muscle (Fig. 2d). Mitochondrial DNA (mtDNA) copy number, and the expression of key transcriptional activators mediating mitochondrial biogenesis—*PGC1α* (*Ppargc1a*), *PGC1β* (*Ppargc1b*) and nuclear respiratory factor 1 (*Nrf1*)—were similar in *Stk25* transgenic vs wild-type muscle (ESM Figs 4, 5).

Our previous studies have shown that the glycogen content was similar in gastrocnemius muscle homogenates of *Stk25* transgenic and wild-type mice [10]. Consistent with these findings, PAS staining in glycogen-rich type IIa and IIx fibres was similar between the genotypes (Fig. 2e).

### Overexpression of STK25 induces lipid accumulation and represses mitochondrial function in myoblasts

**Fig. 3:**
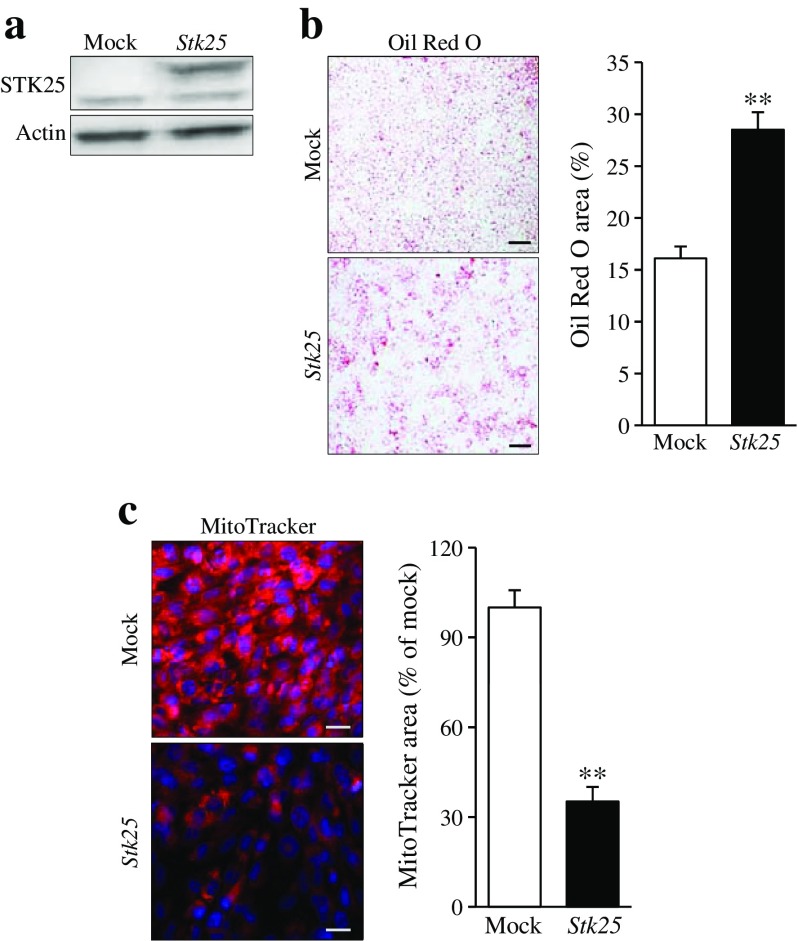
Analysis of lipid accumulation and mitochondrial function in rodent myoblasts overexpressing STK25. L6 cells were transiently transfected with *Stk25* expression plasmid or vector control (mock) and incubated with oleic acid for 24 h. (**a**) Representative western blot with anti-STK25 antibodies; actin was used as a loading control (endogenous STK25 48 kDa, FLAG-tagged STK25 51 kDa). (**b**) Representative cell images stained with Oil Red O (scale bar, 50 μm) and quantification of Oil Red O staining. (**c**) Representative cell images stained with MitoTracker Red (scale bar, 20 μm) and quantification of MitoTracker staining. Data are mean ± SEM from 3–5 wells. **p < 0.01

The global overexpression of STK25 in transgenic mice does not allow us to address whether the impact of STK25 on skeletal muscle lipid metabolism is direct or secondary to the action of STK25 in tissues other than muscle. To study the cell-specific role of STK25 in muscle cells, we transiently transfected the rat myoblast cell line L6 with the *STK25* expression plasmid or an empty control plasmid (mock; Fig. 3a). Subsequent to transfection, the cells were exposed to oleic acid, which efficiently induces steatosis in vitro and thereby mimics the dietary challenge in mice. To analyse lipid deposition, cells were stained with Oil Red O. STK25 overexpression increased lipid accumulation 1.8 ± 0.1-fold based on quantification of Oil Red O staining (Fig. 3b). Notably, staining with MitoTracker Red was 2.8 ± 0.05-fold lower in cells overexpressing STK25 suggesting an impairment of mitochondrial function (Fig. 3c). Consistent with the reduced MitoTracker Red signal, there was a tendency (*p* = 0.09) for a lower rate of β-oxidation in cells overexpressing STK25 (ESM Fig. 6a).

As with the results obtained in *Stk25* transgenic vs wild-type muscle (see above), mtDNA copy number and the expression of key transcriptional activators mediating mitochondrial biogenesis (*PGC1α*, *PGC1β* and *Nrf1*) were largely similar in cells transfected with the *STK25* expression plasmid and vector control (ESM Fig. 6b, c). Our previous studies have shown that in skeletal muscle of *Stk25* transgenic mice fed a high-fat diet, the mRNA expression of carnitine palmitoyltransferase 1 (CPT1), the rate-limiting enzyme in fatty acid oxidation in mitochondria, was significantly reduced [10]. Surprisingly, *Cpt1* mRNA levels were markedly increased in STK25-overexpressing L6 cells (ESM Fig. 6c); while the significance of this observation remains unclear, in view of other data suggesting repressed mitochondrial function.

### Overexpression of STK25 in mice reduces skeletal muscle β-oxidation, while lipid uptake and synthesis remain unaltered

**Fig. 4:**
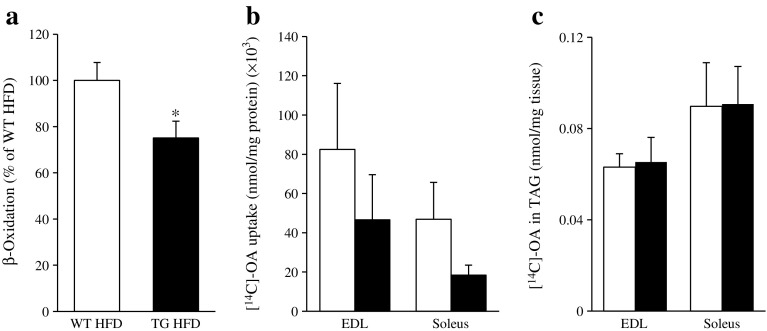
Assessment of lipid metabolism in *Stk25* transgenic and wild-type mice. (**a**) β-oxidation in quadriceps muscle extract. (**b**) Oleic acid uptake and (**c**) triacylglycerol synthesis in isolated EDL and soleus muscle. White bars, wild-type mice fed high-fat diet; black bars, transgenic mice fed high-fat diet. Data are mean ± SEM from 16–18 (**a**) or 11–12 (**b**, **c**) mice per genotype. *p < 0.05. HFD, high-fat diet; OA, oleic acid; TAG, triacylglycerol; TG, transgenic; WT, wild-type

We further characterised lipid metabolism in skeletal muscle of *Stk25* transgenic and wild-type mice fed a high-fat diet ex vivo. Consistent with the reduced MitoTracker Red staining and repressed activity of oxidative metabolism markers, the muscle homogenates of *Stk25* transgenic mice displayed a lower β-oxidation rate (75% of the capacity of wild-type; Fig. 4a). Notably, no significant difference in the level of acylcarnitines, the by-products of incomplete fatty acid oxidation and markers of skeletal muscle insulin resistance, was observed in muscle homogenates comparing the genotypes (ESM Fig. 7).

In addition, we incubated isolated skeletal muscle from *Stk25* transgenic and wild-type mice with radiolabelled oleate and observed that cell-associated radioactivity was not significantly altered between the genotypes, indicating similar fatty acid influx (Fig. 4b). Furthermore, skeletal muscles isolated from both genotypes displayed similar incorporation of oleate into triacylglycerol (Fig. 4c).

### *Stk25* transgenic mice have reduced running performance

**Fig. 5:**
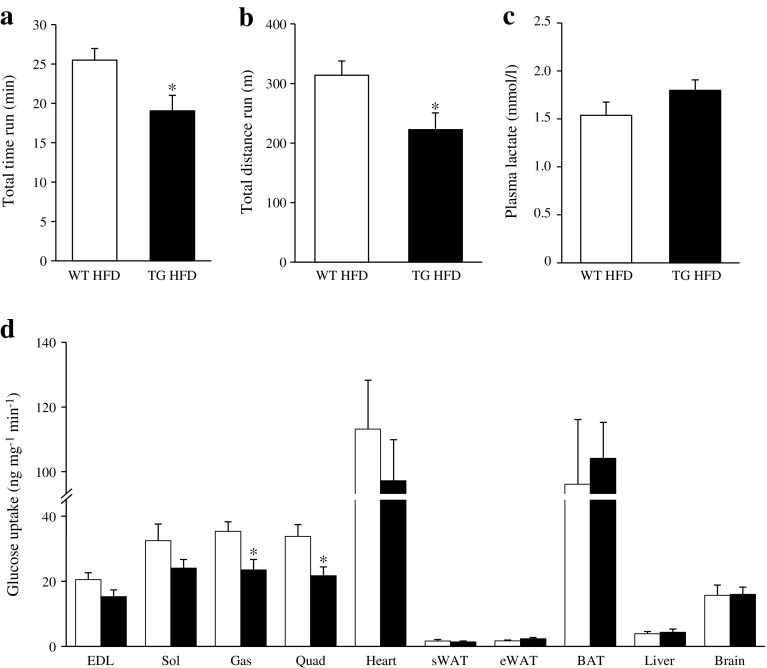
Assessment of endurance running capacity and insulin sensitivity in *Stk25* transgenic and wild-type mice. (**a**) Time and (**b**) distance to fatigue during a treadmill run. (**c**) Plasma lactate levels measured directly after exercise using an l-Lactate Assay Kit (Abcam, Cambridge, UK). (**d**) Insulin-stimulated glucose uptake in individual tissues determined during a euglycaemic–hyperinsulinaemic clamp. White bars, wild-type mice fed high-fat diet; black bars, transgenic mice fed high-fat diet. Data are mean ± SEM from 8–9 mice per genotype. *p < 0.05. BAT, brown adipose tissue; eWAT, epididymal white adipose tissue; Gas, gastrocnemius muscle; HFD, high-fat diet; Quad, quadriceps muscle; Sol, soleus muscle; sWAT, subcutaneous white adipose tissue; TG, transgenic; WT, wild-type

To assess the impact of the disorganised myofibril architecture and fibrosis observed in *Stk25* transgenic muscle on endurance exercise capacity, we next compared the responses of *Stk25* transgenic and wild-type mice fed a high-fat diet to treadmill running. A markedly reduced exercise performance was found in *Stk25* transgenic mice compared with wild-type littermates both in terms of running time and distance to fatigue (Fig. 5a, b). The post-exercise concentration of plasma lactate, a by-product of anaerobic glycolysis, was comparable between the genotypes (Fig. 5c).

### *Stk25* transgenic mice display reduced in vivo insulin-stimulated glucose uptake in skeletal muscle

The observation that STK25 overexpression increased intramyocellular lipid levels and favoured fat storage rather than oxidation in the muscle prompted us to investigate whether these changes would affect skeletal muscle insulin sensitivity. To this end, euglycaemic–hyperinsulinaemic clamp experiments with a glucose tracer were performed in *Stk25* transgenic and wild-type mice fed a high-fat diet. Insulin infusion significantly increased plasma insulin concentration at the end of the clamp for both genotypes (ESM Fig. 8a). There was no difference in glucose infusion rate or blood glucose level at steady state of the clamp comparing the two genotypes (ESM Fig. 8b). Insulin-stimulated glucose uptake was 1.5 ± 0.1-fold and 1.6 ± 0.1-fold lower in gastrocnemius and quadriceps muscles of *Stk25* transgenic mice, respectively, with a similar tendency seen in EDL and soleus muscles (Fig. 5d).

### Global phosphoproteomic analysis of skeletal muscle in *Stk25* transgenic and wild-type mice

**Fig. 6:**
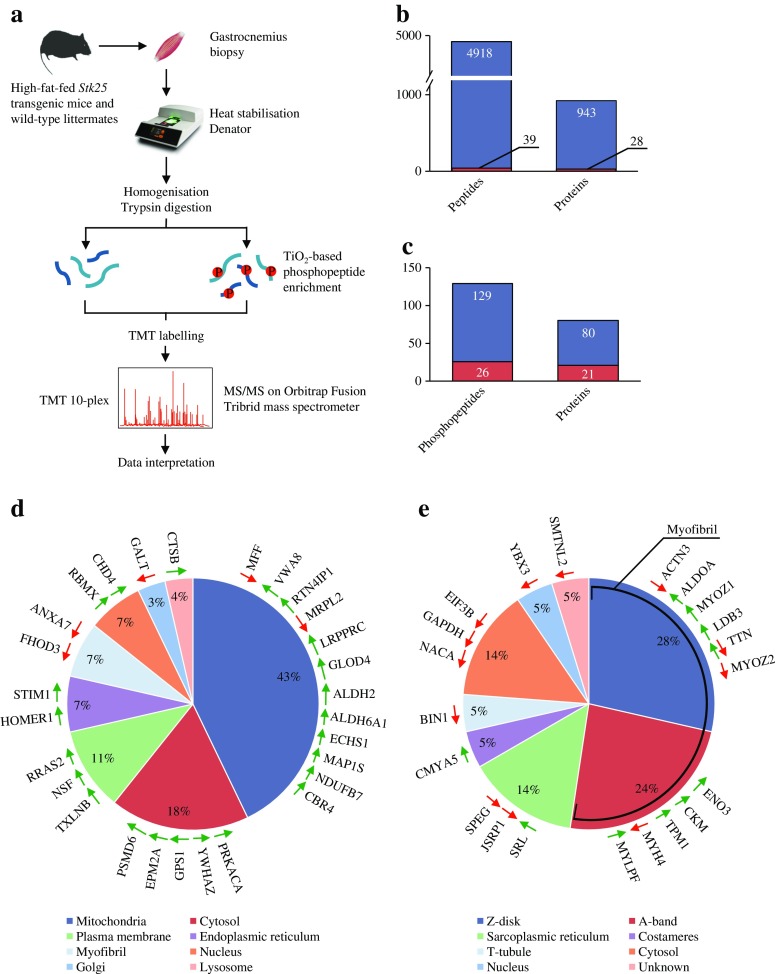
Global quantitative phosphoproteomic analysis in gastrocnemius muscle of *Stk25* transgenic and wild-type mice. (**a**) Experimental design. Summary of the quantified (blue bars) and differentially regulated (red bars) (**b**) proteome and (**c**) phosphoproteome. The subcellular location of the (**d**) differentially expressed and (**e**) differentially phosphorylated proteins was annotated according to the Gene Ontology database [50], NCBI OMIM and/or PubMed. Up- or downregulation is indicated by green and red arrows, respectively

To identify potential downstream mediators of STK25 action in skeletal muscle metabolism in an unbiased manner, global quantitative phosphoproteomic analysis was performed in the gastrocnemius muscle of *Stk25* transgenic and wild-type mice fed a high-fat diet with multiplexed isobaric labelling and phosphopeptide enrichment coupled to tandem MS (MS/MS) (Fig. 6a). The analysis also included quantification of non-phosphorylated peptides to determine possible changes in total protein abundance (Fig. 6a). A total of 4918 distinct peptides and 129 phosphopeptides, corresponding to 943 and 80 unique proteins, respectively, were quantified (Fig. 6b, c). We observed that the abundance of 39 peptides representing 28 unique proteins was differentially regulated, by a factor of 1.15-fold or more, in *Stk25* transgenic muscle relative to the wild-type controls (Fig. 6b, Table 1). Furthermore, we found that the phosphorylation level of 26 peptides derived from 21 proteins was differentially regulated, by a factor of 1.15-fold or more, comparing the genotypes (Fig. 6c, Table 2). No difference in the abundance of the total proteins corresponding to the altered phosphorylation sites was observed, indicating that changes in phosphopeptide levels were a direct result of alterations in their phosphorylation status.

**Table 1:** Differential regulation of the total protein abundance in gastrocnemius skeletal muscle of *Stk25* transgenic and wild-type mice fed a high-fat diet

Accession number	Symbol	Name	*p* value	Transgenic-to-wild-type ratio	Gene function
Q92W1	*Stk25*	Serine/threonine protein kinase 25 (EC 2.7.11.1)	0.0007	12.40	STK25 belongs to the STE20 serine/threonine protein kinase superfamily. It has been shown to be involved in regulation of cell migration, modulation of cell death and control of glucose tolerance and insulin sensitivity
Q8VBT1	*Txlnb*	Taxilin beta	0.03	1.87	The taxilin family is composed of three members in mammals, where taxilin beta is abundantly expressed in the skeletal muscle and heart. Available evidence suggests that taxilin proteins interact with several syntaxin family members and are involved in intracellular vesicle traffic, especially transport of the vesicles delivered to the plasma membrane
Q9Z2Y3	*Homer1*	Homer homologue 1	0.04	1.74	Homer proteins belong to a family of adaptor proteins, which play different roles in cell function, including the regulation of GPCRs and Ca^2+^ homeostasis. HOMER1 associates both with STIM1 and the Cav1.2 alpha1 subunit upon Ca^2+^ store depletion, which indicates a functional role in supporting the interaction between STIM1 and Cav1.2 channels
Q91VT4	*Cbr4*	Carbonyl reductase 4	0.001	1.71	CRB4 and HSD17B8 are the two subunits that make up KAR (EC 1.1.1.100), which catalyses the second step of the mitochondrial fatty acid synthesis pathway
Q9WV02	*Rbmx* (alias *Hnrnpg*)	RNA binding motif protein, X chromosome (alias heterogeneous nuclear ribonucleoprotein G)	0.03	1.62	RBMX is a ubiquitously expressed nuclear glycoprotein implicated in pre-mRNA splicing and in regulation of cellular splicing preferences
Q76LL6	*Fhod3* (alias *Fhos2*)	Formin homology 2 domain containing 3 (alias formin homologue overexpressed in spleen 2)	0.04	−1.59	FHOD3 is a formin family protein, which has actin-organising activity and has been shown to associate with the nestin intermediate filaments
Q03249	*Galt*	Galactose-1-phosphate uridyl transferase (EC 2.7.7.12)	0.04	−1.57	GALT is the second enzyme in the evolutionarily conserved galactose metabolic pathway, and facilitates the simultaneous conversion of uridine diphosphoglucose and galactose-1 phosphate to uridine diphosphogalactose and glucose-1 phosphate It has been shown that GALT-deficient mice accumulate galactitol and galactonate in heart and skeletal muscle
Q6PDQ2	*Chd4*	Chromodomain helicase DNA binding protein 4	0.01	1.53	CHD4 is a chromatin-remodelling enzyme that has been reported to regulate DNA damage responses
Q6PCP5	*Mff*	Mitochondrial fission factor	0.01	−1.51	MFF is anchored to the mitochondrial outer membrane through a C-terminal transmembrane domain. Depletion of MFF promotes mitochondrial fusion, resulting in an interconnected tubular network of mitochondria; in contrast, exogenous expression of MFF induces extensive mitochondrial fragmentation
P05132	*Prkaca* (alias *Pkaca*)	Protein kinase, cAMP dependent, catalytic, alpha (alias protein kinase A catalytic subunit alpha)	0.001	1.50	Most of the effects of cAMP in the eukaryotic cell are mediated through the phosphorylation of target proteins on Ser or Thr residues by PKA (EC 2.7.11.11). Inactive PKA is a tetramer composed of 2 regulatory and 2 catalytic subunits. The cooperative binding of 4 molecules of cAMP dissociates the enzyme in a regulatory subunit dimer and 2 free active catalytic subunits. In the human, 3 catalytic subunits (PRKACA, PRKACB and PRKACG) have been identified. PKA anchoring proteins (AKAPs) modulate PKA-dependent phosphorylation by tethering this kinase to a specific subcellular location
P10605	*Ctsb*	Cathepsin B	0.01	1.50	Cathepsins are the ubiquitously expressed major lysosomal proteases and they primarily determine the proteolytic capacity of lysosomes. Cathepsin B has been implicated in signalling pathways of apoptosis, liver fibrosis and muscle wasting
Q8CC88	*Vwa8*	Von Willebrand factor A domain-containing protein 8	0.0003	1.49	VWA8 is a protein of unknown function with a high mitochondrial localisation prediction
P63101	*Ywhaz* (alias *14-3-3ζ*)	Tyrosine 3-monooxygenase/tryptophan 5-monooxygenase activation protein, zeta polypeptide (alias 14-3-3 protein zeta)	0.049	1.49	YWHAZ is involved in the import of precursor proteins into mitochondria. The expression of YWHAZ appears to be upregulated in a number of human tumours, suggesting that this protein may exhibit oncogenic properties. YWHAZ is reported to interact with several targets such as with IRS1 and PKB (Akt1), although the functional significance of these interactions remains unclear
Q99LD4	*Gps1* (alias *Csn1*)	G protein pathway suppressor 1 (alias COP9/signalosome complex subunit)	0.02	1.40	GPS1 is one of the 8 subunits of the COP9 signalosome (CSN). The cullin deneddylation activity of CSN requires all its subunits and regulates cullin-RING ligases, thereby controlling ubiquitination of a large number of proteins
Q9WUA5	*Epm2a*	Epilepsy, progressive myoclonic epilepsy, type 2 gene alpha (alias laforin)	0.03	1.40	EPM2A is, by sequence, a member of the atypical dual specificity protein phosphatase subfamily, but it has been shown to dephosphorylate polysaccharides including glycogen and amylopectin. Loss-of-function mutations in the *EPM2A* gene cause the fatal neurodegenerative disorder in humans, Lafora disease, characterised by increased glycogen phosphorylation and the formation of abnormal deposits of glycogen-like material called Lafora bodies
Q924D0	*Rtn4ip1* (alias *Nimp*)	Reticulon 4 interacting protein 1 (alias NOGO-interacting mitochondrial protein)	0.01	1.36	RTN4IP1 is a highly conserved and ubiquitously expressed novel mitochondrial ubiquinol oxydo-reductase. Mutations in *RTN4IP1* have been associated with a deficit of mitochondrial respiratory complex I and IV activities, and increased susceptibility to UV light
P46460	*Nsf*	*N*-ethylmaleimide sensitive fusion protein	0.03	1.36	NSF was the first protein found to play a key role in eukaryotic trafficking. In concert with the adaptor protein SNAP, NSF disassembles the SNARE complex into individual proteins upon ATP hydrolysis. By disassembling post-fusion SNARE complexes, NSF is essential for maintaining pools of fusion-ready individual SNARE proteins that mediate membrane fusion in a variety of cellular processes
Q9D773	*Mrpl2*	Mitochondrial ribosomal protein L2	0.04	−1.36	Mitochondria have their own translation system for production of proteins essential for oxidative phosphorylation. MRPL2 is one of the protein components of mitochondrial ribosomes that are encoded by the nuclear genome
Q6PB66	*Lrpprc*	Leucine-rich PPR-motif containing	0.04	1.34	LRPPRC regulates stability of mitochondrial DNA-encoded mRNAs. Inactivation of LRPPRC impairs mitochondrial respiration and reduces ATP production caused mainly by an ATP synthase deficiency
P62071	*Rras2* (alias *Tc21*)	Related RAS viral (R-Ras) oncogene homologue 2	0.04	1.34	RRAS2 is the ubiquitously expressed member of the R-Ras family of Ras-related proteins. Deregulated RRAS2 activity has been suggested to contribute to human oncogenesis
Q9CPV4	*Glod4*	Glyoxalase domain containing 4	0.01	1.25	GLOD4 is a mitochondrial protein implicated in metabolic detoxification; however, the exact function of GLOD4 is not known
P70302	*Stim1*	Stromal interaction molecule 1	0.049	1.24	STIM1 is a transmembrane protein, mainly located to the membrane of the endoplasmic reticulum. STIM1 is considered a key element in the activation of store-operated Ca^2+^ (SOC) entry by mediating the communication of the filling state of the Ca^2+^ stores to the plasma membrane channels
Q99JI4	*Psmd6*	Proteasome (prosome, macropain) 26S subunit, non-ATPase, 6	0.01	1.23	In eukaryotes, protein turnover is almost entirely accomplished by a single enzyme, the 26S proteasome. PSMD6 acts as a regulatory subunit of the 26S proteasome, and is probably involved in the ATP-dependent degradation of ubiquitinated proteins
Q07076	*Anxa7* (alias *Anx7*)	Annexin A7 (alias annexin-7, synexin)	0.05	−1.21	ANXA7 is a calcium-dependent membrane-binding protein that regulates membrane fusion and acts as a voltage-dependent calcium channel. ANXA7 participates in cellular Ca^2+^ signalling and has been implicated if different cellular processes such as spherocytosis, inflammatory myopathies, cardiac remodelling, regulation of cell survival and tumour growth as well as in excitation–contraction coupling in skeletal muscle
P47738	*Aldh2*	Aldehyde dehydrogenase 2, mitochondrial (EC 1.2.1.3)	0.02	1.21	Aldehyde dehydrogenases catalyse the conversion of reactive aldehydes to carboxylates. Mitochondrial ALDH2 is known to oxidise acetaldehyde produced from ethanol into acetate
Q9EQ20	*Aldh6a1* (alias *Mmsdh*)	Aldehyde dehydrogenase family 6, subfamily A1 [alias methylmalonate-semialdehyde dehydrogenase (acylating), mitochondrial] (EC 1.2.1.27)	0.03	1.19	The *Aldh6a1* gene encodes mitochondrial methylmalonate semialdehyde dehydrogenase, an enzyme that catalyses the irreversible oxidative decarboxylation of malonate and methylmalonate semialdehydes to acetyl- and propionyl-CoA, respectively. This activity is part of the valine and pyrimidine catabolic pathways
Q8BH95	*Echs1* (alias *Sceh*)	Enoyl coenzyme A hydratase, short chain, 1, mitochondrial (alias enoyl-CoA hydratase 1) (EC 4.2.1.17)	0.01	1.17	ECHS1 catalyses the second step in mitochondrial fatty acid oxidation
Q8C052	*Map1s* (alias *Map8, C19ORF5*)	Microtubule-associated protein 1S (alias microtubule-associated protein 8)	0.03	1.16	The ubiquitously distributed MAP1S has been implicated in microtubule dynamics and mitotic abnormalities, mitotic cell death and autophagy regulation. MAP1S is an interactive partner of mitochondrion-associated LRPPRC and RASSF1 as well as ND1 and COX-I. MAP1S deficiency in mice causes an accumulation of large swollen mitochondria indicative of a potential defect in mitophagy, while accumulation of MAP1S was associated with irreversible aggregation of mitochondria in mammalian cells
Q9CR61	*Ndufb7*	NADH dehydrogenase (ubiquinone) 1 beta subcomplex 7	0.02	1.15	NDUFB7 is a eukaryotic complex I subunit that resides in the mitochondrial intermembrane space. NADH:ubiquinone oxidoreductase (complex I) catalyses the first step in oxidative phosphorylation. It couples the oxidation of NADH to the reduction of ubiquinone and the translocation of protons across the inner membrane

A ratio of 1.15-fold serves as the threshold for differential regulation. The functions of the differentially expressed proteins were annotated according to Gene Ontology database [50], NCBI OMIM and/or PubMed

AKAP, PKA-anchoring protein; COX-I, cytochrome c oxidase subunit I; COP9, constitutive photomorphogenesis 9; GPCR, G-protein coupled receptor; HSD17B8, hydroxysteroid (17-beta) dehydrogenase 8; KAR, 3-ketoacyl-acyl carrier protein (ACP) reductase; LRPPRC, leucine-rich PPR-motif-containing; ND1, NADH dehydrogenase subunit 1; PKA, cAMP dependent protein kinase; PKB, protein kinase B; RASSF1, RAS association (RalGDS/AF-6) domain family member 1; SNAP, soluble *N*-ethylmaleimide-sensitive factor attachment protein; SNARE, soluble *N*-ethylmaleimide-sensitive factor attachment protein receptor; STIM1, stromal interaction molecule 1

**Table 2:** Differential regulation of the phosphorylation pattern in gastrocnemius skeletal muscle of *Stk25* transgenic and wild-type mice fed a high-fat diet

Accession number	Symbol	Name	Phospho-rylation site	*p* value	Transgenic-to-wild-type ratio	Gene function
A2ASS6	*Ttn*	Titin	S13904^a^	0.003	2.45	Titin is a giant muscle protein that spans from Z-disk to M-disk and plays a key role in muscle assembly, force transmission at the Z-disk, and maintenance of resting tension in the I-band region
			S814^a^	0.02	−1.46	
			S22390^a^	0.05	1.36	
P97457	*Mylpf* (alias *Mlc2*)	Myosin light chain, phosphorylatable, fast skeletal muscle	S15^a^	0.001	2.29	MYLPF is controlled by phosphorylation and modulates muscle contraction properties
Q7TQ48	*Srl*	Sarcalumenin	S464^a^	0.04	1.88	Sarcalumenin is a Ca^2+^ binding protein localised to the sarcoplasmic reticulum that is considered to be important in the excitation–contraction–relaxation cycle in skeletal muscle cells
P07310	*Ckm* (alias *M-ck*)	Creatine kinase, muscle (EC 2.7.3.2)	T166^a^	0.02	1.81	Creatine kinase catalyses the reversible transfer of a phosphate from phosphocreatine to ADP, maintaining intracellular ATP levels
			T327^a^	0.02	1.31	
P05064	*Aldoa*	Aldolase A, fructose-bisphosphate (EC 4.1.2.13)	S276^a^	0.05	1.73	ALDOA is a glycolytic enzyme that catalyses the reversible conversion of fructose-1,6-bisphosphate to glyceraldehyde 3-phosphate and dihydroxyacetone phosphate
Q8CI12	*Smtnl2*	Smoothelin-like 2	S98^a^	0.04	−1.67	SMTNL2 is a functionally uncharacterised protein from the SMTN family with notably high expression in skeletal muscle
			S339^a^	0.03	−1.34	
P58771	*Tpm1*	Tropomyosin 1, alpha	S283^a^	0.005	1.67	Tropomyosin 1 represents a critical myofilament protein in the Ca^2+^ regulation of actin–myosin interaction, striated muscle contraction, and relaxation. TPM1 is the predominant isoform in the mammalian heart and fast skeletal muscle
P21550	*Eno3*	Enolase 3, beta muscle (EC 4.2.1.11)	S40^a^	0.001	1.64	Muscle-specific ENO3 is a glycolytic enzyme that resides at the M-disk and converts the glycolytic intermediate 2-phospho-d-glycerate to phosphoenolpyruvate
Q9JJW5	*Myoz2* (alias *Cs-1*)	Myozenin 2 (alias calsarcin-1)	S116^a^	0.00005	1.61	Myozenins are calcineurin-interacting proteins that also bind to ACTN2 and LDB3 at the Z-disk, suggesting a role in modulating calcium–calcineurin-dependent signalling
Q70KF4	*Cmya5*	Cardiomyopathy associated 5 (alias myospryn)	S769^a^	0.02	1.61	CMYA5 is a multifunctional desmin-binding protein that also associates with PKA. CMYA5 is suggested to participate in the subcellular targeting of PKA activity in striated muscle
Q62407	*Speg*	SPEG complex locus	S1177^a^	0.03	−1.55	SPEG is a protein localised to the sarcoplasmic reticulum that interacts with myotubularin; SPEG deficiency causes CNM in humans
Q8JZQ9	*Eif3b*	Eukaryotic translation initiation factor 3, subunit B	S75^a^	0.05	−1.33	EIF3B is part of the EIF3 complex
Q9JKB3	*Ybx3* (alias *Csda*)	Y box protein 3 (alias cold-shock domain protein A)	S328^a^	0.03	−1.55	YBX3 is a transcriptional repressor, which is highly expressed in skeletal muscle, where it specifically regulates myogenin expression
Q9JK37	*Myoz1* (alias *Cs-2*)	Myozenin 1 (alias calsarcin-2)	S111	0.004	−1.48	Myozenins are calcineurin-interacting proteins that also bind to ACTN2 and LDB3 at the Z-disk, suggesting a role in modulating calcium–calcineurin-dependent signalling
O88990	*Actn3*	Actinin alpha 3	S601^a^	0.001	−1.38	ACTN3 is localised to the Z-disk, where it helps to anchor the actin filaments and regulates contractile properties of the muscle
Q5SX39	*Myh4* (alias *MhcIIb*)	Myosin, heavy polypeptide 4, skeletal muscle (alias myosin heavy chain IIb)	T258^a^	0.05	−1.32	Skeletal muscle fibres are classified by speed of contraction, where expression of MYH4 defines the fast-twitch glycolytic type IIb fibres
			S1339	0.02	−1.24	
P16858	*Gapdh*	Glyceraldehyde-3-phosphate dehydrogenase (EC 1.2.1.12)	T209^a^	0.02	−1.27	GAPDH catalyses an important energy-yielding step in carbohydrate metabolism, the reversible oxidative phosphorylation of glyceraldehyde-3-phosphate in the presence of inorganic phosphate and NAD
O08539	*Bin1* (alias *Amph2*)	Bridging integrator 1 (alias amphiphysin II)	T308^a^	0.001	−1.25	BIN1 is localised in skeletal muscle at deep sarcolemmal invaginations, the T-tubules, implicated in excitation–contraction coupling; mutations in *BIN1* have been described in CNM in humans
Q3MI48	*Jsrp1*	Junctional sarcoplasmic reticulum protein 1	S228^a^	0.03	−1.23	JSRP1 is an integral protein constituent of the skeletal muscle sarcoplasmic reticulum membrane, involved in the development and maintenance of skeletal muscle strength
P70670	*Naca*	Nascent polypeptide-associated complex alpha polypeptide	S1489^a^	0.02	−1.21	NACA, exclusively found in skeletal and heart muscle, is suggested to be involved in skeletal muscle development, homeostasis and regeneration, although the underlying mechanisms remain unclear
Q9JKS4	*Ldb3* (alias *Zasp*)	LIM domain binding 3 (alias Z-band alternatively spliced PDZ motif-containing protein)	S98^a^	0.02	1.20	LDB3 is a PDZ-LIM domain binding factor that plays an important role in maintaining the structural integrity of the striated muscle Z-disk. LDB3 binds to myozenin and ACTN2

A ratio of 1.15-fold serves as the threshold for differential regulation. The functions of the differentially phosphorylated proteins were annotated according to Gene Ontology database [50], NCBI OMIM and/or PubMed

^a^Phosphosites annotated in PhosphoSitePlus [32]

ACTN2, actinin alpha 2; CNM, centronuclear myopathy; NAD, nicotinamide adenine dinucleotide; PKA, cAMP dependent protein kinase

Assessment of the known cellular localisation of the differentially expressed proteins revealed a marked enrichment of the targets located to mitochondria (Fig. 6d, Table 1). Among these candidates, carbonyl reductase 4 (CBR4), an enzyme in the mitochondrial fatty acid synthesis pathway [24], reticulon 4 interacting protein 1 (RTN4IP1), enoyl coenzyme A hydratase, short chain, 1 (ECHS1), and NADH dehydrogenase (ubiquinone) 1 beta subcomplex 7 (NDUFB7)—all involved in mitochondrial oxidation [25–27]—as well as two mitochondrial aldehyde dehydrogenases (ALDH2 and ALDH6A1 [28, 29]) were upregulated in transgenic muscle. In addition, mitochondrial fission factor (MFF) and microtubule-associated protein 1S (MAP1S), which control mitochondrial morphology by regulating dynamic fission and fusion process [30, 31], were differentially expressed comparing the genotypes. Apart from these candidates with relatively well-known functions, several additional proteins with poorly described roles in mitochondria were differentially expressed in transgenic muscle, including von Willebrand factor A domain-containing protein 8 (VWA8) and glyoxalase domain-containing 4 (GLOD4).

Analysis of proteins containing differentially regulated phosphosites, on the other hand, revealed an enrichment of the targets with known location in the Z-disk, where actin-containing thin filaments from neighbouring sarcomeres overlap cross-linked by alpha-actinin, and in the myosin-containing A-band of the sarcomere (Fig. 6e, Table 2). These myofibril-associated candidates included proteins with well-characterised roles in regulation of contractile properties of the skeletal muscle, such as titin (TTN), myosin regulatory light chain (MYLPF), creatine kinase (CKM), tropomyosin 1, alpha (TPM1), actinin alpha 3 (ACTN3) and myosin heavy chain II beta (MYH4 or MHCIIB), as well as proteins with less defined roles, such as myozenin 1 and 2 (MYOZ1 and 2) and LIM domain binding 3 (LDB3). Interestingly, the phosphorylation of the three enzymes in the glycolytic pathway—aldolase A, fructose-bisphosphate (ALDOA), enolase 3, beta muscle (ENO3) and glyceraldehyde-3-phosphate dehydrogenase (GAPDH)—was altered in *Stk25* transgenic compared with wild-type muscle. Of the 26 differentially regulated phosphosites, 24 were annotated in PhosphoSitePlus [32]. However, the functional implication of the phosphorylation at these sites has only been described for TPM1, where phosphorylation at a single Ser-283 residue has been associated with increased Ca^2+^ activated ATPase activity [33] and regulation of Ca^2+^ sensitivity [34].

Notably, our previous studies have shown that overexpression of STK25 repressed lipolysis in mouse and human hepatocytes, which probably contributed to the increased lipid storage observed in these cells [7, 8]. The global phosphoproteomic analysis by MS/MS did not detect any lipases above the level of quantification in the skeletal muscle. However, we also measured the skeletal muscle mRNA and protein levels of two lipases (ATGL and HSL) by quantitative real-time PCR and western blot, respectively, and observed no differences between the genotypes (ESM Fig. 9). Recently, the levels of the lipid droplet binding proteins Perilipin 2 (PLIN2 also known as adipose differentiation-related protein [ADRP]) and Perilipin 3 (PLIN3), as well as the activity of AMP-activated protein kinase (AMPK), have been implicated in the regulation of lipid content in muscle cells [35, 36]. PLIN3 was quantified by MS/MS analysis without any alterations in total protein abundance observed between the genotypes, while PLIN2 and AMPK were below the level of quantification. However, we found that the skeletal muscle mRNA of *Plin2* and *Plin3*, and protein abundance of PLIN2 measured by western blot, were not changed when comparing the genotypes (ESM Fig. 9). Furthermore, our previous studies have shown that AMPK activity, measured by AMPKα Thr172 phosphorylation, was similar when comparing the skeletal muscle of *Stk25* transgenic and wild-type mice [10].

## Discussion

**Fig. 7:**
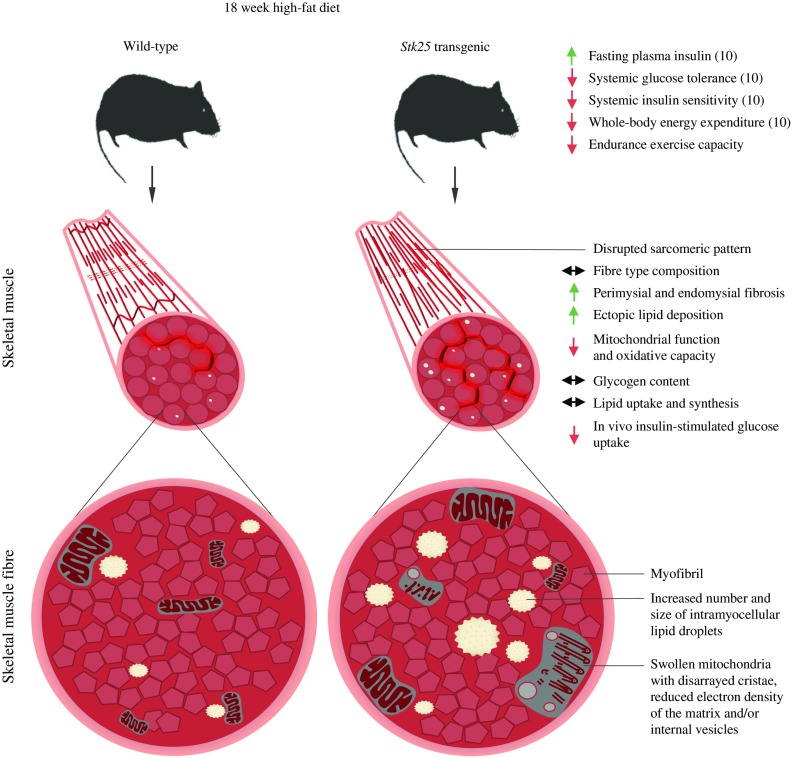
Schematic illustration of metabolic responses at the whole body level as well as in skeletal muscle fibres of *Stk25* transgenic mice vs wild-type littermates. Up- or downregulation is indicated by green and red arrows, respectively

Insulin resistance in skeletal muscle is a major and early feature in the pathogenesis of type 2 diabetes [37]. In this study, we provide compelling evidence that overexpression of protein kinase STK25 in transgenic mice challenged with a high-fat diet increases myocellular lipid storage and impairs skeletal muscle mitochondrial function as well as sarcomeric ultrastructure, thereby reducing endurance exercise capacity and repressing muscle insulin responsiveness (Fig. 7). Furthermore, we found enhanced lipid accumulation and impaired mitochondrial function in rodent myoblasts overexpressing STK25, demonstrating an autonomous action of STK25 in muscle cells. These results are consistent with our previous studies showing that *Stk25* transgenic mice fed a high-fat diet develop aggravated whole body glucose intolerance and insulin resistance compared with wild-type littermates [10].

The main finding of this study is the substantially increased presence of ultrastructural abnormalities of both subsarcolemmal and intermyofibrillar mitochondria in *Stk25* transgenic skeletal muscle, which was associated with reduced MitoTracker Red staining, lower abundance of oxidative metabolism markers and suppressed β-oxidation. Previous studies using TEM have demonstrated alterations in mitochondrial morphology in skeletal muscle of humans and rodent models with insulin resistance and type 2 diabetes [38–40]. In addition, reduced in vivo mitochondrial oxidative capacity has been reported in the skeletal muscle of patients with type 2 diabetes [41–43]. Furthermore, studies in healthy elderly individuals and insulin-resistant offspring of parents with type 2 diabetes have demonstrated that repressed mitochondrial function may predispose these individuals to intramyocellular lipid accumulation and insulin resistance [44]. Moreover, coordinated reduction in the expression of genes involved in mitochondrial function and oxidative phosphorylation was reported in skeletal muscle from type 2 diabetes patients, but also from individuals with insulin resistance but normal glucose tolerance [45, 46]. Based on this evidence, impaired mitochondrial function observed in *Stk25* transgenic muscle probably contributed to increased myocellular lipid storage and development of skeletal muscle insulin resistance.

We further explored the underlying mechanisms involved in the regulation of the mitochondrial structure and activity by STK25. We failed to detect any accumulation of STK25 protein in skeletal muscle mitochondria (ESM Fig. 2c), and therefore, STK25 is likely to control mitochondrial function indirectly through the regulation of abundance and/or phosphorylation pattern of, as yet, unknown mitochondrial target proteins. Interestingly, global phosphoproteomic analysis revealed that the two key regulators of mitochondrial fusion and fission process were differentially expressed, with MFF protein levels decreased and MIP1S protein levels increased, in *Stk25* transgenic vs wild-type muscle. MFF knockdown in mammalian cells resulted in an interconnected tubular network of mitochondria, whereas MFF overexpression stimulated mitochondrial fission [31, 47]. Accumulation of MAP1S, on the other hand, was associated with irreversible aggregation of mitochondria [48]. Based on this evidence, we speculate that altered protein abundance of MFF and MAP1S may be one underlying mechanism for impaired mitochondrial morphology in *Stk25* transgenic muscle.

We also found disorganised myofibril architecture and pronounced fibrosis in *Stk25* transgenic muscle, which probably contributed to reduced endurance exercise capacity. This was not observed in wild-type muscle. Notably, global phosphoproteomic analysis revealed a marked enrichment of the differentially phosphorylated proteins located in the myofibril, in particular to the Z-disk and A-band of the sarcomere, in *Stk25* transgenic vs wild-type muscle. However, at present it is not known whether these changes have contributed to the disrupted sarcomere organisation in transgenic muscle. Interestingly, recent evidence shows that perturbations in skeletal muscle sarcomere ultrastructure in individuals with heart failure and type 2 diabetes can be improved by stimulating mitochondrial function [49], which supports a close functional connection between mitochondrial alterations and muscle damage, and suggests that mitochondrial abnormalities observed in *Stk25* transgenic muscle may have contributed to altered myofibril architecture.

The metabolic changes in the skeletal muscle of *Stk25* transgenic mice are consistent with our previous observations in mouse and human liver cells, where STK25 overexpression in conditions of excess dietary fuels increased intrahepatocellular lipid deposition while repressing mitochondrial function and insulin sensitivity [7–9]. Taken together, these findings suggest that STK25 may regulate the shift in the metabolic balance from lipid use to lipid storage in several tissues prone to diabetic damage, contributing to the pathogenesis of whole body insulin resistance and type 2 diabetes.

In this study, we characterised the skeletal muscle phenotype of *Stk25* transgenic and wild-type mice challenged with a high-fat diet in order to mimic conditions in high-risk individuals. Notably, our previous investigations have shown that liver lipid deposition and whole body insulin sensitivity were not significantly altered comparing *Stk25* transgenic vs wild-type mice fed regular chow [9, 10]. Moreover, we found that skeletal muscle triacylglycerol content and fibrosis were not increased in *Stk25* transgenic mice fed regular chow compared with corresponding wild-type littermates (ESM Fig. 10). These data suggest that overexpression of STK25 leads to significant metabolic alterations in mice only after a dietary challenge.

To date, it remains unknown whether any physiological situations occur in which the STK25 protein abundance is enhanced to a level observed in the *Stk25* transgenic muscle, which is a limitation of the animal model. Notably, our findings in *Stk25* transgenic muscle are reciprocal to our previous observations of increased β-oxidation and improved insulin action in STK25-deficient myoblasts [11] as well as reduced lipid accumulation and enhanced insulin sensitivity in the skeletal muscle of *Stk25* ^-/-^ mice fed a high-fat diet [6], reinforcing the physiological validity of the results.

In light of the current epidemic of type 2 diabetes, research aimed at understanding the interplay between intramyocellular lipid storage, mitochondrial energetics, and insulin action in skeletal muscle is of utmost importance for the development of new therapeutic strategies. This study provides several layers of evidence that STK25 is an interesting new mediator in the interconnected metabolic network controlling skeletal muscle insulin sensitivity, and that the development of STK25 antagonists for therapeutic applications in type 2 diabetes and related metabolic disease is warranted.

## Electronic supplementary material

Below is the link to the electronic supplementary material.

ESM 1 (PDF 1700 kb)

## Abbreviations

COX: Cytochrome c oxidase
EDL: Extensor digitorum longus
H-E: Haematoxylin-eosin
HSL: Hormone-sensitive lipase
LC: Liquid chromatography
MAP1S: Microtubule-associated protein 1S
MFF: Mitochondrial fission factor
MHC: Myosin heavy chain
PAS: Periodic acid–Schiff
SDH: Succinate dehydrogenase
STK25: Serine/threonine protein kinase 25
TEM: Transmission electron microscopy

## References

[1] Anstee QM, Targher G, Day CP (2013). Progression of NAFLD to diabetes mellitus, cardiovascular disease or cirrhosis. Nat Rev Gastroenterol Hepatol.

[2] Perseghin G, Scifo P, De Cobelli F (1999). Intramyocellular triglyceride content is a determinant of in vivo insulin resistance in humans: a 1H-13C nuclear magnetic resonance spectroscopy assessment in offspring of type 2 diabetic parents. Diabetes.

[3] Goodpaster BH, Wolf D (2004). Skeletal muscle lipid accumulation in obesity, insulin resistance, and type 2 diabetes. Pediatr Diabetes.

[4] DeFronzo RA, Jacot E, Jequier E, Maeder E, Wahren J, Felber JP (1981). The effect of insulin on the disposal of intravenous glucose. Results from indirect calorimetry and hepatic and femoral venous catheterization. Diabetes.

[5] Thompson BJ, Sahai E (2015). MST kinases in development and disease. J Cell Biol.

[6] Amrutkar M, Cansby E, Chursa U (2015). Genetic disruption of protein kinase STK25 ameliorates metabolic defects in a diet-induced type 2 diabetes model. Diabetes.

[7] Amrutkar M, Cansby E, Nunez-Duran E (2015). Protein kinase STK25 regulates hepatic lipid partitioning and progression of liver steatosis and NASH. FASEB J.

[8] Amrutkar M, Kern M, Nunez-Duran E (2016). Protein kinase STK25 controls lipid partitioning in hepatocytes and correlates with liver fat content in humans. Diabetologia.

[9] Amrutkar M, Chursa U, Kern M (2016). STK25 is a critical determinant in nonalcoholic steatohepatitis. FASEB J.

[10] Cansby E, Amrutkar M, Manneras Holm L (2013). Increased expression of STK25 leads to impaired glucose utilization and insulin sensitivity in mice challenged with a high-fat diet. FASEB J.

[11] Nerstedt A, Cansby E, Andersson CX (2012). Serine/threonine protein kinase 25 (STK25): a novel negative regulator of lipid and glucose metabolism in rodent and human skeletal muscle. Diabetologia.

[12] Osada S, Izawa M, Saito R (1997). YSK1, a novel mammalian protein kinase structurally related to Ste20 and SPS1, but is not involved in the known MAPK pathways. Oncogene.

[13] Pombo CM, Bonventre JV, Molnar A, Kyriakis J, Force T (1996). Activation of a human Ste20-like kinase by oxidant stress defines a novel stress response pathway. EMBO J.

[14] Preisinger C, Short B, De Corte V (2004). YSK1 is activated by the Golgi matrix protein GM130 and plays a role in cell migration through its substrate 14-3-3zeta. J Cell Biol.

[15] Voss K, Stahl S, Schleider E (2007). CCM3 interacts with CCM2 indicating common pathogenesis for cerebral cavernous malformations. Neurogenetics.

[16] Matsuki T, Matthews RT, Cooper JA (2010). Reelin and stk25 have opposing roles in neuronal polarization and dendritic Golgi deployment. Cell.

[17] Fidalgo M, Fraile M, Pires A, Force T, Pombo C, Zalvide J (2010). CCM3/PDCD10 stabilizes GCKIII proteins to promote Golgi assembly and cell orientation. J Cell Sci.

[18] Nogueira E, Fidalgo M, Molnar A (2008). SOK1 translocates from the Golgi to the nucleus upon chemical anoxia and induces apoptotic cell death. J Biol Chem.

[19] Zhou J, Shao Z, Kerkela R (2009). Serine 58 of 14-3-3zeta is a molecular switch regulating ASK1 and oxidant stress-induced cell death. Mol Cell Biol.

[20] De Paepe B, De Bleecker JL, Van Coster R (2009). Histochemical methods for the diagnosis of mitochondrial diseases. Curr Protoc Hum Genet.

[21] Anderberg C, Cunha SI, Zhai Z (2013). Deficiency for endoglin in tumor vasculature weakens the endothelial barrier to metastatic dissemination. J Exp Med.

[22] Wu Q, Ortegon AM, Tsang B, Doege H, Feingold KR, Stahl A (2006). FATP1 is an insulin-sensitive fatty acid transporter involved in diet-induced obesity. Mol Cell Biol.

[23] Bruce CR, Brolin C, Turner N (2007). Overexpression of carnitine palmitoyltransferase I in skeletal muscle in vivo increases fatty acid oxidation and reduces triacylglycerol esterification. Am J Physiol Endocrinol Metab.

[24] Chen Z, Kastaniotis AJ, Miinalainen IJ, Rajaram V, Wierenga RK, Hiltunen JK (2009). 17beta-hydroxysteroid dehydrogenase type 8 and carbonyl reductase type 4 assemble as a ketoacyl reductase of human mitochondrial FAS. FASEB J.

[25] Angebault C, Guichet PO, Talmat-Amar Y (2015). Recessive mutations in RTN4IP1 cause isolated and syndromic optic neuropathies. Am J Hum Genet.

[26] Sakai C, Yamaguchi S, Sasaki M, Miyamoto Y, Matsushima Y, Goto Y (2015). ECHS1 mutations cause combined respiratory chain deficiency resulting in Leigh syndrome. Hum Mutat.

[27] Szklarczyk R, Wanschers BF, Nabuurs SB, Nouws J, Nijtmans LG, Huynen MA (2011). NDUFB7 and NDUFA8 are located at the intermembrane surface of complex I. FEBS Lett.

[28] Nakashima Y, Ohsawa I, Nishimaki K (2014). Preventive effects of chlorella on skeletal muscle atrophy in muscle-specific mitochondrial aldehyde dehydrogenase 2 activity-deficient mice. BMC Complement Altern Med.

[29] Marcadier JL, Smith AM, Pohl D (2013). Mutations in ALDH6A1 encoding methylmalonate semialdehyde dehydrogenase are associated with dysmyelination and transient methylmalonic aciduria. Orphanet J Rare Dis.

[30] Xie R, Nguyen S, McKeehan K, Wang F, McKeehan WL, Liu L (2011). Microtubule-associated protein 1S (MAP1S) bridges autophagic components with microtubules and mitochondria to affect autophagosomal biogenesis and degradation. J Biol Chem.

[31] Otera H, Wang C, Cleland MM (2010). Mff is an essential factor for mitochondrial recruitment of Drp1 during mitochondrial fission in mammalian cells. J Cell Biol.

[32] Hornbeck PV, Zhang B, Murray B, Kornhauser JM, Latham V, Skrzypek E (2015). PhosphoSitePlus, 2014: mutations, PTMs and recalibrations. Nucleic Acids Res.

[33] Nixon BR, Liu B, Scellini B (2013). Tropomyosin Ser-283 pseudo-phosphorylation slows myofibril relaxation. Arch Biochem Biophys.

[34] Lu X, Heeley DH, Smillie LB, Kawai M (2010). The role of tropomyosin isoforms and phosphorylation in force generation in thin-filament reconstituted bovine cardiac muscle fibres. J Muscle Res Cell Motil.

[35] Kleinert M, Parker BL, Chaudhuri R (2016). mTORC2 and AMPK differentially regulate muscle triglyceride content via Perilipin 3. Mol Metab.

[36] Bosma M, Hesselink MK, Sparks LM (2012). Perilipin 2 improves insulin sensitivity in skeletal muscle despite elevated intramuscular lipid levels. Diabetes.

[37] DeFronzo RA, Tripathy D (2009). Skeletal muscle insulin resistance is the primary defect in type 2 diabetes. Diabetes Care.

[38] Ritov VB, Menshikova EV, He J, Ferrell RE, Goodpaster BH, Kelley DE (2005). Deficiency of subsarcolemmal mitochondria in obesity and type 2 diabetes. Diabetes.

[39] Kelley DE, He J, Menshikova EV, Ritov VB (2002). Dysfunction of mitochondria in human skeletal muscle in type 2 diabetes. Diabetes.

[40] Liu Y, Turdi S, Park T (2013). Adiponectin corrects high-fat diet-induced disturbances in muscle metabolomic profile and whole-body glucose homeostasis. Diabetes.

[41] Phielix E, Schrauwen-Hinderling VB, Mensink M (2008). Lower intrinsic ADP-stimulated mitochondrial respiration underlies in vivo mitochondrial dysfunction in muscle of male type 2 diabetic patients. Diabetes.

[42] Schrauwen-Hinderling VB, Kooi ME, Hesselink MK (2007). Impaired in vivo mitochondrial function but similar intramyocellular lipid content in patients with type 2 diabetes mellitus and BMI-matched control subjects. Diabetologia.

[43] Petersen KF, Dufour S, Befroy D, Garcia R, Shulman GI (2004). Impaired mitochondrial activity in the insulin-resistant offspring of patients with type 2 diabetes. N Engl J Med.

[44] Morino K, Petersen KF, Shulman GI (2006). Molecular mechanisms of insulin resistance in humans and their potential links with mitochondrial dysfunction. Diabetes.

[45] Mootha VK, Lindgren CM, Eriksson KF (2003). PGC-1alpha-responsive genes involved in oxidative phosphorylation are coordinately downregulated in human diabetes. Nat Genet.

[46] Patti ME, Butte AJ, Crunkhorn S (2003). Coordinated reduction of genes of oxidative metabolism in humans with insulin resistance and diabetes: potential role of PGC1 and NRF1. Proc Natl Acad Sci U S A.

[47] Toyama EQ, Herzig S, Courchet J (2016). Metabolism. AMP-activated protein kinase mediates mitochondrial fission in response to energy stress. Science.

[48] Liu L, Xie R, Yang C, McKeehan WL (2009). Dual function microtubule- and mitochondria-associated proteins mediate mitotic cell death. Cell Oncol.

[49] Taub PR, Ramirez-Sanchez I, Ciaraldi TP (2013). Perturbations in skeletal muscle sarcomere structure in patients with heart failure and type 2 diabetes: restorative effects of (-)-epicatechin-rich cocoa. Clin Sci (Lond).

[50] Ashburner M, Ball CA, Blake JA (2000). Gene ontology: tool for the unification of biology. The Gene Ontology Consortium. Nat Genet.

