# Supplementary material for: Overexpression of protein kinase STK25 in mice exacerbates ectopic lipid accumulation, mitochondrial dysfunction and insulin resistance in skeletal muscle
Source: Diabetologia. 2016 Dec 16;60(3):553–67. doi: 10.1007/s00125-016-4171-5 (PMC6518105; doi:10.1007/s00125-016-4171-5)
Supplement: Supplementary file 1 — (PDF 1700 kb) [file 125_2016_4171_MOESM1_ESM.pdf]

**ESM Methods**

*In Vivo Assessment of Exercise Performance*

Endurance exercise capacity was assessed by treadmill running (Columbus Instruments, Columbus, OH, US) until the mice reached fatigue. This was defined as spending ≥20 s at the base of the treadmill despite manual encouragement. Initial speed was 10 m/min and the velocity was increased by 2 m/min every 5 min. Once treadmill speed reached 22 m/min, this speed was maintained until fatigue. Before experimental testing, mice were subjected to a 3-day familiarization protocol that consisted of progressively increasing the intensity and duration of treadmill running. All experiments were performed in between 10:00 and 13:00 h.

*In Vivo Assessment of Insulin Sensitivity*

Insulin sensitivity was measured by euglycemic-hyperinsulinemic clamp. Surgery and glucose and insulin infusion were performed as previously described [1] using an insulin infusion rate of 7 mU/min/kg. When the steady-state was reached (at t=90 min), a bolus of 2-deoxy-D-[$^{14}$C]-glucose (1.5 µCi; PerkinElmer, Waltham, MA, US) was injected by the jugular vein. Blood was sampled at 93, 96, 100, 105, 110, 120, 130, and 150 min postinjection. Mice were then killed, and tissues were analysed for glucose uptake as previously described [2]. Circulating glucose and insulin concentrations were measured using an Accu-Chek glucometer (Roche Diagnostics, Basel, Switzerland) and Ultrasensitive Mouse Insulin ELISA Kit (Chrystal Chem Inc., Downers Grove, IL, US), respectively. The glucose infusion rate (GIR) was calculated as the amount of glucose infused per kilogram body weight per minute.

*Liquid Chromatography Mass Spectrometry (LC-MS) Analysis*

Fresh gastrocnemius muscle samples were heat stabilized using a Denator instrument (Stabilizor™ T1, Uppsala, Sweden) according to the manufacturer's instructions. Tissue was homogenized in lysis buffer [50 mmol/l triethylammonium bicarbonate (TEAB) (Fluka, Sigma Aldrich, Stockholm, Sweden), 2% SDS, and PhosSTOP phosphatase inhibitor cocktail tablets (Roche, Bromma, Sweden)], using a FastPrep®-24 instrument (MP Biomedicals, Täby, Sweden). Protein concentration was determined with Pierce™ 660 nm Protein Assay Kit (Thermo Scientific, Stockholm, Sweden). Samples were processed using the filter-aided sample preparation method [3]. 100 µg protein was used for total proteome comparison and 1 mg protein per sample was used for phosphoproteomics. Proteins were treated with dithiothreitol (DTT) and methyl methanethiosulfonate (MMTS), double digested with trypsin (Sequencing Grade Modified Trypsin, Promega, Nacka, Sweden) in 1% sodium deoxycholate (SDC) and 20 mmol/l TEAB at 37°C. Phosphoproteomic samples were desalted using C18 Strata-X™ Column (Phenomenex Inc., Torrance, CA, US) after precipitation of SDC, processed with Pierce™ $TiO_2$ Phosphopeptide Enrichment and Clean-Up Kit (Thermo Scientific), and pH adjusted to alkaline condition. All samples were subjected to isobaric mass tagging reagent TMT (Thermo Scientific) according to the manufacturer's instructions. TMT sets were combined, acidified and desalted using C18 Strata-X™ Column or Pierce™ C18 Spin Columns (Thermo Scientific), and reconstituted in 0.1% formic acid and 3% acetonitrile.

Each TMT10-plex set was analysed twice (most and least intense precursor ions) on an Orbitrap Fusion Tribrid mass spectrometer interfaced to an Easy-nLC 1000 (Thermo Scientific). Peptides were separated in a column (300x0.075 mm I.D.) packed with 1.8 μm Reprosil-Pur C18-AQ particles (Dr. Maisch, Ammerbuch-Entringen, Germany) using an acetonitrile gradient in 0.2% formic acid, during 100 min. MS1 scans were performed at 120000 resolution, m/z range 350-1500, followed by MS2 analysis [collision induced dissociation (CID) at 35%, for identification] of selected precursor

ions and MS3 [high-energy collision induced dissociation (HCD) at 55%, for quantification] of several MS2 fragments in parallel. Data were analysed by using Proteome Discoverer version 1.4 (Thermo Scientific) with the Mascot search engine (Matrix Science, London, UK) using the *Mus musculus* SwissProt Database (version February 2015), peptide tolerance of 5 ppm and MS/MS tolerance of 500 millimass units (mmu). Tryptic peptides were accepted with zero missed cleavage. The detected peptide threshold was set to 1% false discovery rate by searching against a reversed database.

**References**

[1] Cansby E, Amrutkar M, Manneras Holm L, et al. (2013) Increased expression of STK25 leads to impaired glucose utilization and insulin sensitivity in mice challenged with a high-fat diet. FASEB J 27: 3660-3671

[2] Burcelin R, Dolci W, Thorens B (2000) Glucose sensing by the hepatoportal sensor is GLUT2-dependent: in vivo analysis in GLUT2-null mice. Diabetes 49: 1643-1648

[3] Wisniewski JR, Zougman A, Nagaraj N, Mann M (2009) Universal sample preparation method for proteome analysis. Nat Methods 6: 359-362

**ESM Table 1.** List of antibodies used for immunofluorescence and Western blot analysis

| Type | Antibody name and catalogue number | Working dilution | Company |
|---|---|---|---|
| Primary antibody | anti-laminin 2 alpha (#ab11576) | 1:1000 | Abcam (Cambridge, UK) |
| | anti-actin (#sc-1616) | 1:1000 | Santa Cruz Biotechnology (Santa Cruz, CA, US) |
| | anti-myosin heavy chain I (MHCI; #BA-F8) | 1:200 | Developmental Studies Hybridoma Bank (Iowa City, IA, US) |
| | anti-myosin heavy chain IIa (MHCIIa; #SC-71) | 1:200 | Developmental Studies Hybridoma Bank |
| | anti-myosin heavy chain IIb (MHCIIb; #BF-F3) | 1:200 | Developmental Studies Hybridoma Bank |
| | anti-myosin heavy chain IIx (MHCIIx; #6H1) | 1:200 | Developmental Studies Hybridoma Bank |
| | anti-STK25 (#NBP1-32670) | 1:300 | Novus Biologicals (Littleton, CO, US) |
| | anti-STK25 (YSK1; #sc-6865) | | Santa Cruz Biotechnology |
| | anti-perilipin 2 (PLIN2; #20R-AP002) | 1:1000 | Fitzgerald Industries International (Concord, MA, US) |
| | anti-hormone sensitive lipase (HSL; #4107) | 1:1000 | Cell Signaling Technology (Boston, MA, US) |
| | anti-adipose triglyceride lipase (ATGL; #sc-67355) | 1:1000 | Santa Cruz Biotechnology |
| Secondary antibody | donkey anti-mouse IgM (#715166020) | 1:1000 | Jackson ImmunoResearch Laboratories (West Grove, PA, US) |
| | donkey anti-rabbit IgG (#A21207) | 1:1000 | Thermo Fisher Scientific (Waltham, MA, US) |
| | donkey anti-goat (#sc-2020) | 1:1000 | Santa Cruz Biotechnology |
| | goat anti-rabbit IgG (#7074S) | 1:1000 | Cell Signaling technology |
| | rabbit anti-guinea pig IgG (#P0141) | 1:1000 | Dako Agilent Technologies (Glostrup, Denmark) |
| | goat anti-rabbit IgG (#A11008) | 1:1000 | Thermo Fisher Scientific |
| | goat anti-rat IgG (#A11007) | 1:1000 | Thermo Fisher Scientific |
| | rabbit anti-mouse IgG (#A11059) | 1:1000 | Thermo Fisher Scientific |

**ESM Table 2.** Sequences of custom-designed primers (forward and reverse) and probes used for quantitative real-time PCR

| **Gene** | | **Sequence (5’-3’)** |
|---|---|---|
| *Ppargc1α* | Forward | CGCAACATGCTCAAGCCA |
| | Reverse | TTAGGCCTGCAGTTCCAGAGAG |
| | Probe | CCAAATGACCCCAAGGGTTCCCC |
| *Ppargc1β* | Forward | GTGGACGAGCTTTCACTGCTA |
| | Reverse | CAGAGCTTGCTGTTGGGGA |
| | Probe | CAGAAGCTCCTCCTGGCCACATCC |
| *Nrf1* | Forward | GCTGATGGAGAGGTGGAACAA |
| | Reverse | GGCTTCTGCCAGTGATGCTA |
| | Probe | TGACCATCCAGACGACGCAAGCA |

# ESM Figure 1

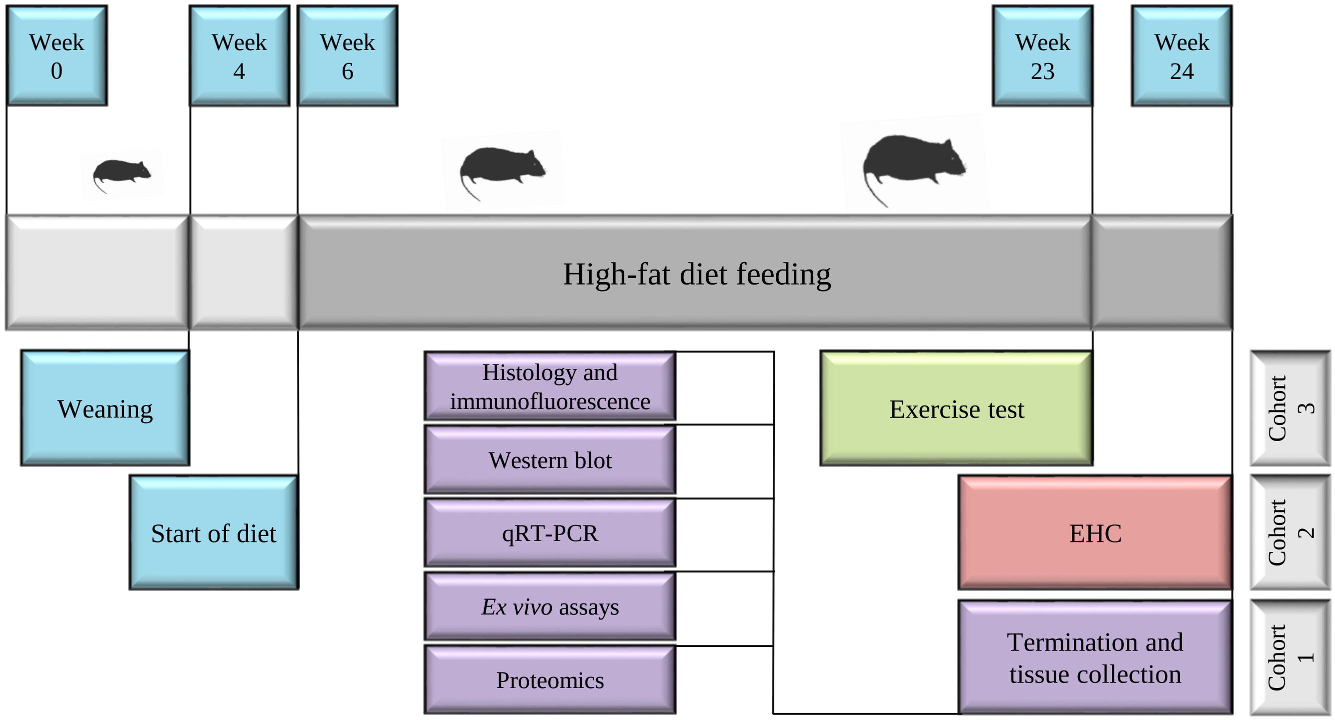


**ESM Figure 1. Schematic presentation of measurements performed in three cohorts of high-fat-fed *Stk25* transgenic and wild-type mice.** EHC, euglycemic-hyperinsulinemic clamp; qRT-PCR, quantitative real-time PCR.

# ESM Figure 2

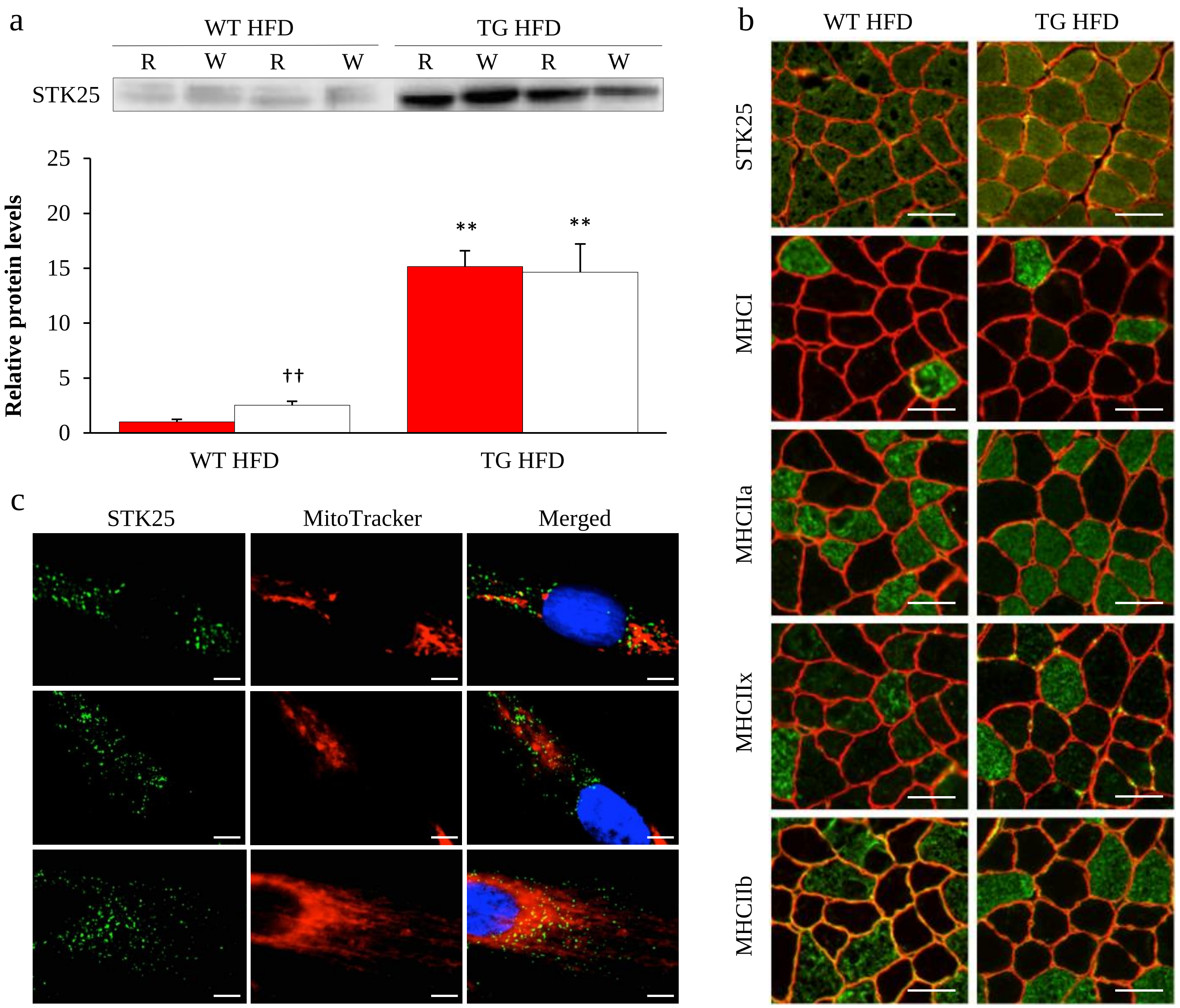


**ESM Figure 2. Analysis of STK25 protein in gastrocnemius skeletal muscle of high-fat-fed *Stk25* transgenic and wild-type mice and primary human muscle cells**. (a) Protein lysates of the red and white part of the gastrocnemius muscle were analysed by Western blot using antibodies specific for STK25. Protein levels were analysed by densitometry and shown as bar histograms. The level of STK25 in the wild-type red gastrocnemius muscle is set to 1. Representative Western blot is shown. Red bars, red part of gastrocnemius muscle; white bars, white part of gastrocnemius muscle. Data are mean ± SEM from 6 mice per genotype. (b) Representative immunofluorescence images of gastrocnemius muscle double-stained with antibodies for STK25, MHC type I, IIa, IIx or IIb (green), and laminin (red). Scale bar, 50 µm. (c) Representative immunofluorescence images of primary human muscle cells double-stained with antibodies for STK25 and MitoTracker Red; nuclei stained with DAPI (blue). The primary human muscle cells were maintained in F-10 Nut Mix (Ham; Gibco) including 4.5 g/l (25 mmol/l) glucose and 1% (vol./vol.) penicillin/ streptomycin (Gibco), supplemented with 20% FBS (Gibco). The cells were differentiated for 11 days in MEM Alpha medium (Gibco) including 1% (vol./vol.) fungizone (Gibco) and 1% (vol./vol.) penicillin/ streptomycin (Gibco), supplemented with 2% (vol./vol.) horse serum (Gibco). Scale bar, 15 µm. $^{**}p < 0.01$ comparing *Stk25* transgenic versus wild-type muscle. $^{††}p < 0.01$ comparing red versus white gastrocnemius in wild-type mice. HFD, high-fat diet; TG, transgenic; WT, wild-type.

# ESM Figure 3

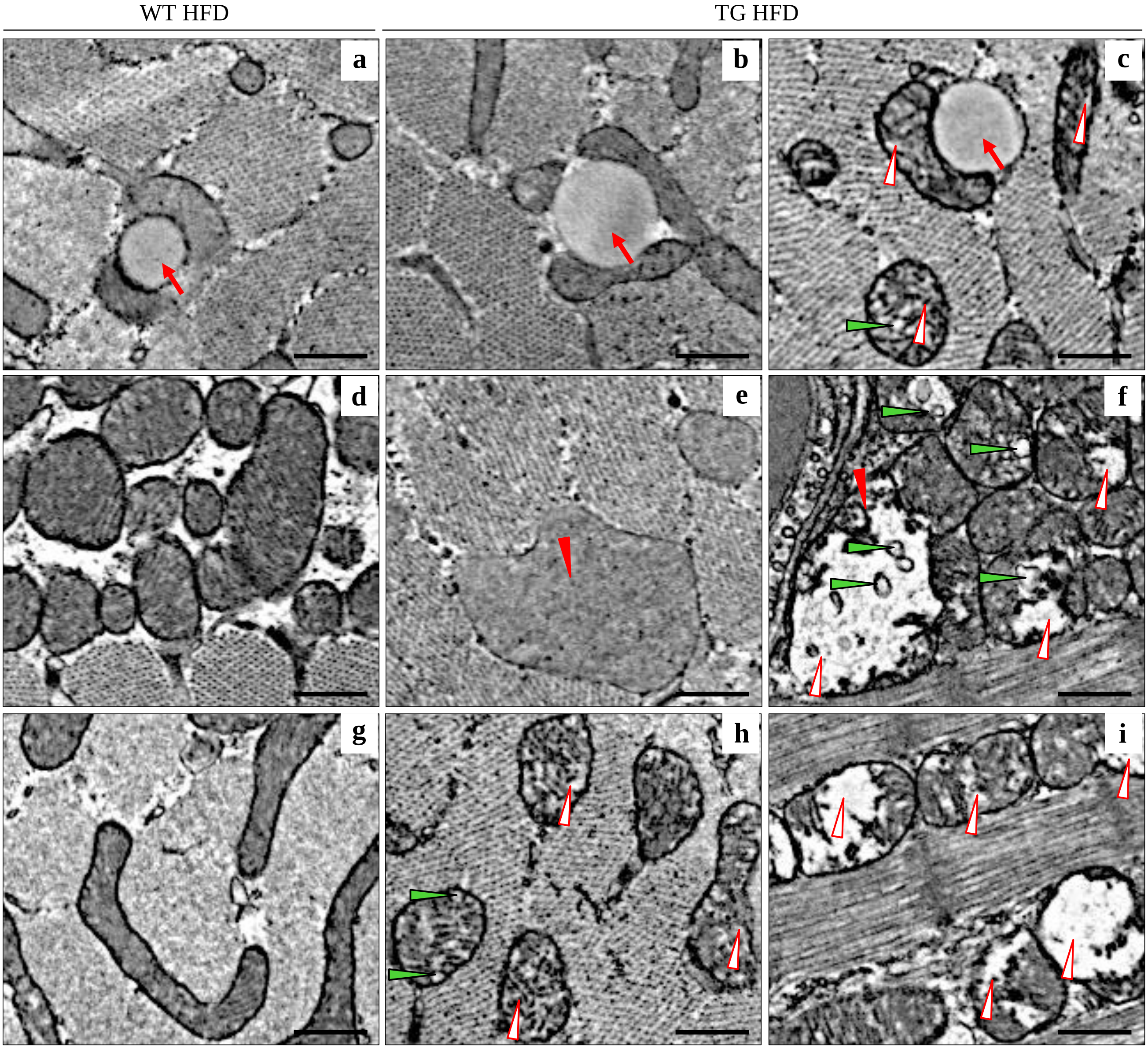


**ESM Figure 3. Representative electron micrographs of gastrocnemius skeletal muscle of high-fat-fed *Stk25* transgenic and wild-type mice.** The cross-sections show lipid droplets (red arrows) and mitochondria, which are swollen (red arrowhead), display disarrayed cristae and reduced electron density of the matrix (open arrowhead), and internal vesicles (green arrowhead). Scale bar, 2 μm. HFD, high-fat diet; TG, transgenic; WT, wild-type.

# ESM Figure 4

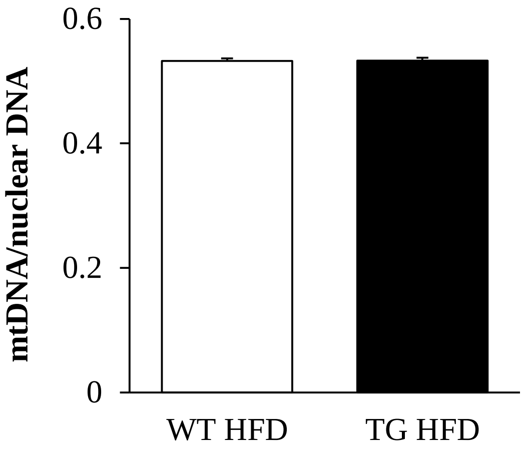


**ESM Figure 4. Measurement of mtDNA copy number in gastrocnemius skeletal muscle of high-fat-fed *Stk25* transgenic and wild-type mice.** Relative mtDNA copy number was calculated as the ratio of a mitochondrial-encoded gene (*COX1*, forward 5′-ACTATACTACTACTAACAGACCG-3′, reverse 5′-GGTTCTTTTTTTCCGGAGTA-3′) to a nuclear-encoded gene (*cyclophilin A*, forward 5′-ACACGCCATAATGGCACTGG-3′, reverse 5′-CAGTCTTGGCAGTGCAGAT-3′) DNA levels, determined by quantitative real-time PCR. Data are mean ± SEM from 9-10 mice per genotype. HFD, high-fat diet; TG, transgenic; WT, wild-type.

# ESM Figure 5

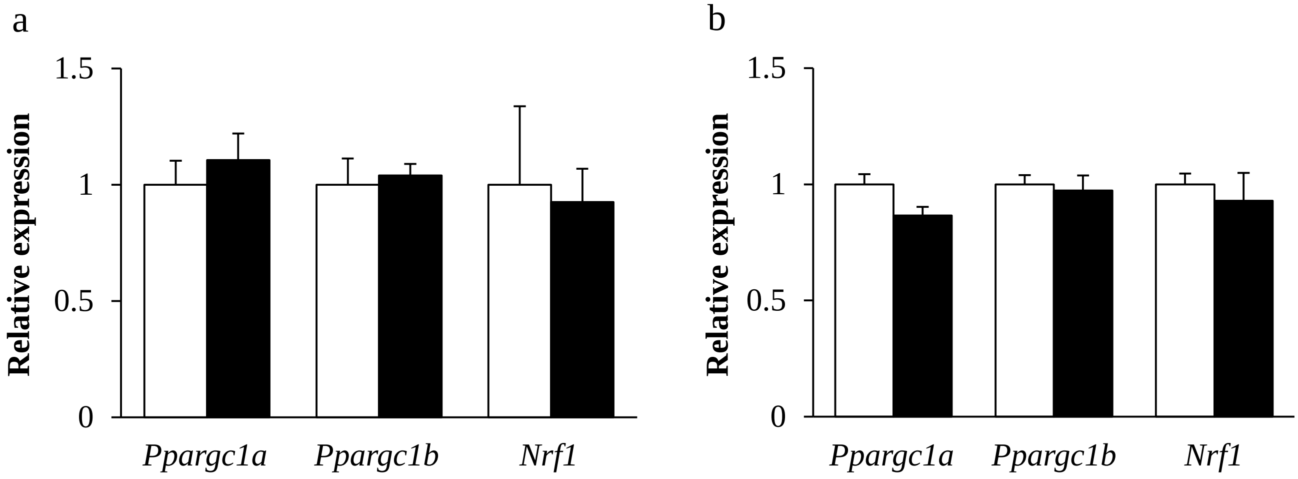


**ESM Figure 5. Measurement of mRNA expression of key transcriptional activators mediating mitochondrial biogenesis in gastrocnemius skeletal muscle of high-fat-fed *Stk25* transgenic and wild-type mice.** Relative mRNA expression of *PGC1α* (*Ppargc1a*), *PGC1β* (*Ppargc1b*), and *Nrf1* in the red (a) and white (b) part of the gastrocnemius muscle was assessed by quantitative real-time PCR using custom-designed primers and probes (see ESM Table 2 for sequences). The expression level of each gene in wild-type mice is set to 1. White bars, high-fat-fed wild-type mice; black bars, high-fat-fed transgenic mice. Data are mean ± SEM from 10-12 mice per genotype.

# ESM Figure 6

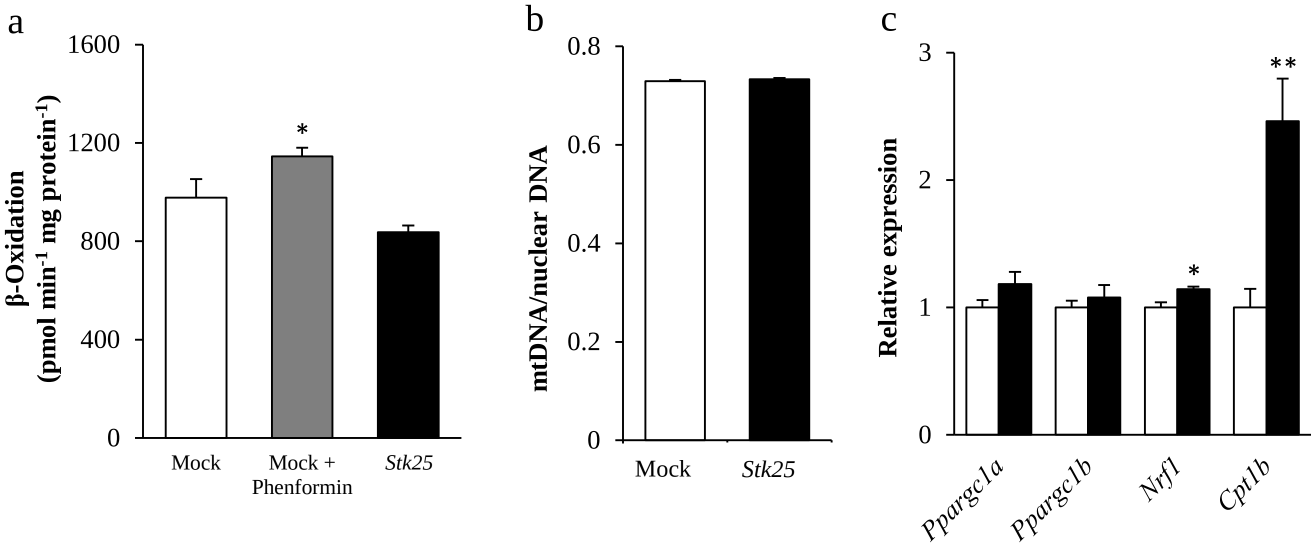


**ESM Figure 6. Measurement of β-oxidation, mtDNA copy number, and mRNA expression in rodent myoblasts overexpressing STK25.** L6 cells were transiently transfected with *STK25* expression plasmid or vector control (mock) and incubated with oleic acid for 24 h. (a) Measurement of β-oxidation; phenformin, which stimulates fatty acid oxidation via activation of AMPK, has been included as a reference substance. (b) Relative mtDNA copy number was calculated as the ratio of a mitochondrial-encoded gene (cytochrome b, forward: 5'-AACGCCAACCCTAGACAACC-3', reverse: 5'-GAGATGTTAGATGGGGCGGG-3') to a nuclear-encoded gene β-actin (forward: 5'-CCACCATGTACCCAGGCATT-3', reverse: 5'-CGGACTCATCGTACTCCTGC-3') DNA levels, determined by quantitative real-time PCR. (c) Relative mRNA expression of *PGC1α (Ppargc1a)*, *PGC1β (Ppargc1b)*, *Nrf1*, and *Cpt1b* was assessed by quantitative real-time PCR using commercially available TaqMan Probe (*Cpt1b*; Assay-on-Demand; Applied Biosystems) or custom-designed primers and probes (see ESM Table 2 for sequences). White bars, cells transfected with vector control (mock); black bars, cells transfected with *STK25* expression plasmid. (b-c) The expression level of each gene in cells transfected with vector control is set to 1. Data are mean ± SEM from 8-12 wells. *$p < 0.05$; **$p < 0.01$.

# ESM Figure 7

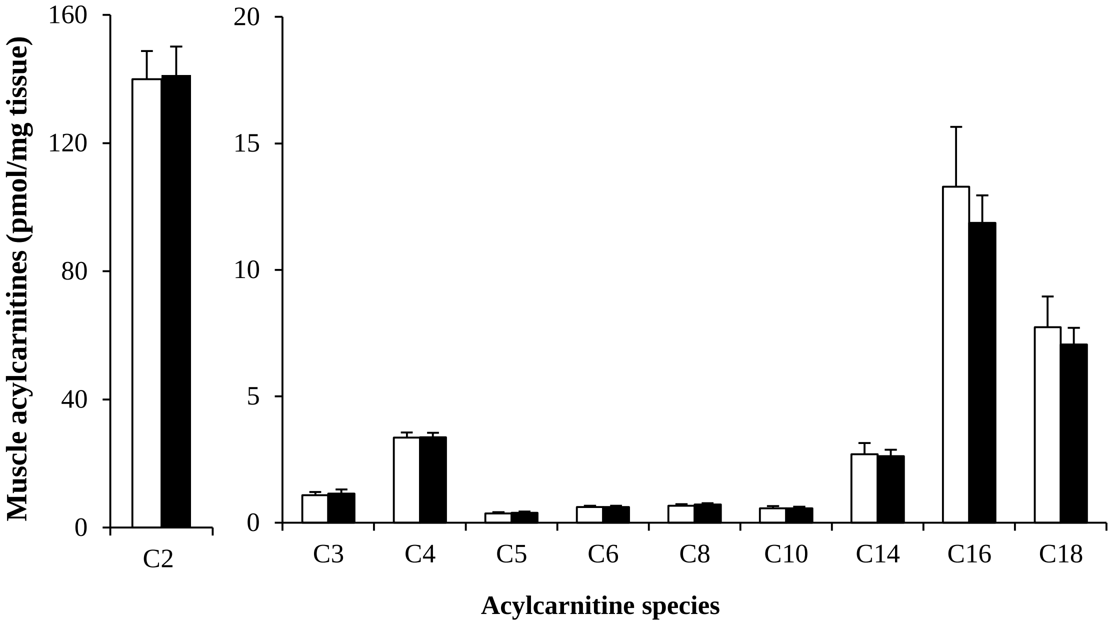


**ESM Figure 7. Acylcarnitine levels in gastrocnemius skeletal muscle of high-fat-fed *Stk25* transgenic and wild-type mice.** Acylcarnitines were extracted in methanol using a Precellys 24 instrument (Bertin Technologies, Montigny-le-Bretonneux, France) and analysed using hydrophilic interaction liquid chromatography tandem mass spectrometry (HILIC-MS/MS). White bars, high-fat-fed wild-type mice; black bars, high-fat-fed transgenic mice. Data are mean ± SEM from 12 mice per genotype.

# ESM Figure 8

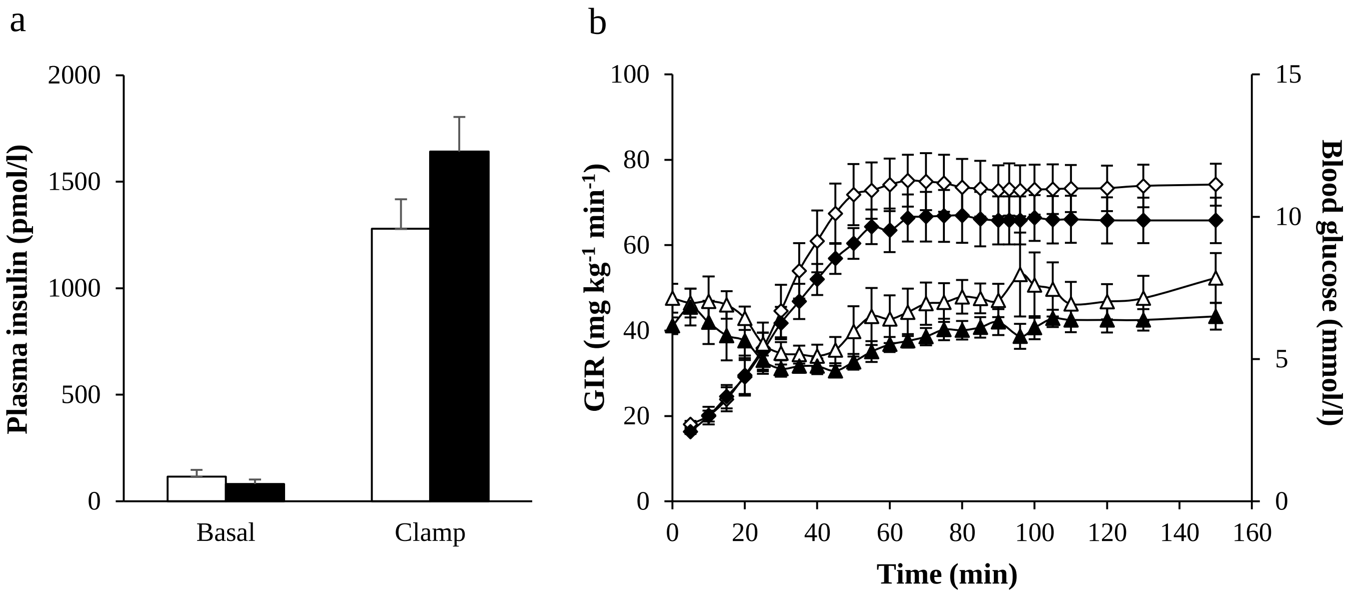


**ESM Figure 8. Plasma insulin and glucose level as well as glucose infusion rate during an euglycemic-hyperinsulinemic clamp in high-fat-fed *Stk25* transgenic and wild-type mice.** Plasma insulin concentration (a), glucose infusion rate and blood glucose concentration (b) determined during an euglycemic-hyperinsulinemic clamp. For (a), white bars, high-fat-fed wild-type mice; black bars, high-fat-fed transgenic mice; for (b), white rhombs, glucose infusion rate in high-fat-fed wild-type mice; black rhombs, glucose infusion rate in high-fat-fed transgenic mice; white triangles, glucose levels in high-fat-fed wild-type mice; black triangles, glucose levels in high-fat-fed transgenic mice. Data are mean ± SEM from 8 mice per genotype. GIR, glucose infusion rate.

# ESM Figure 9

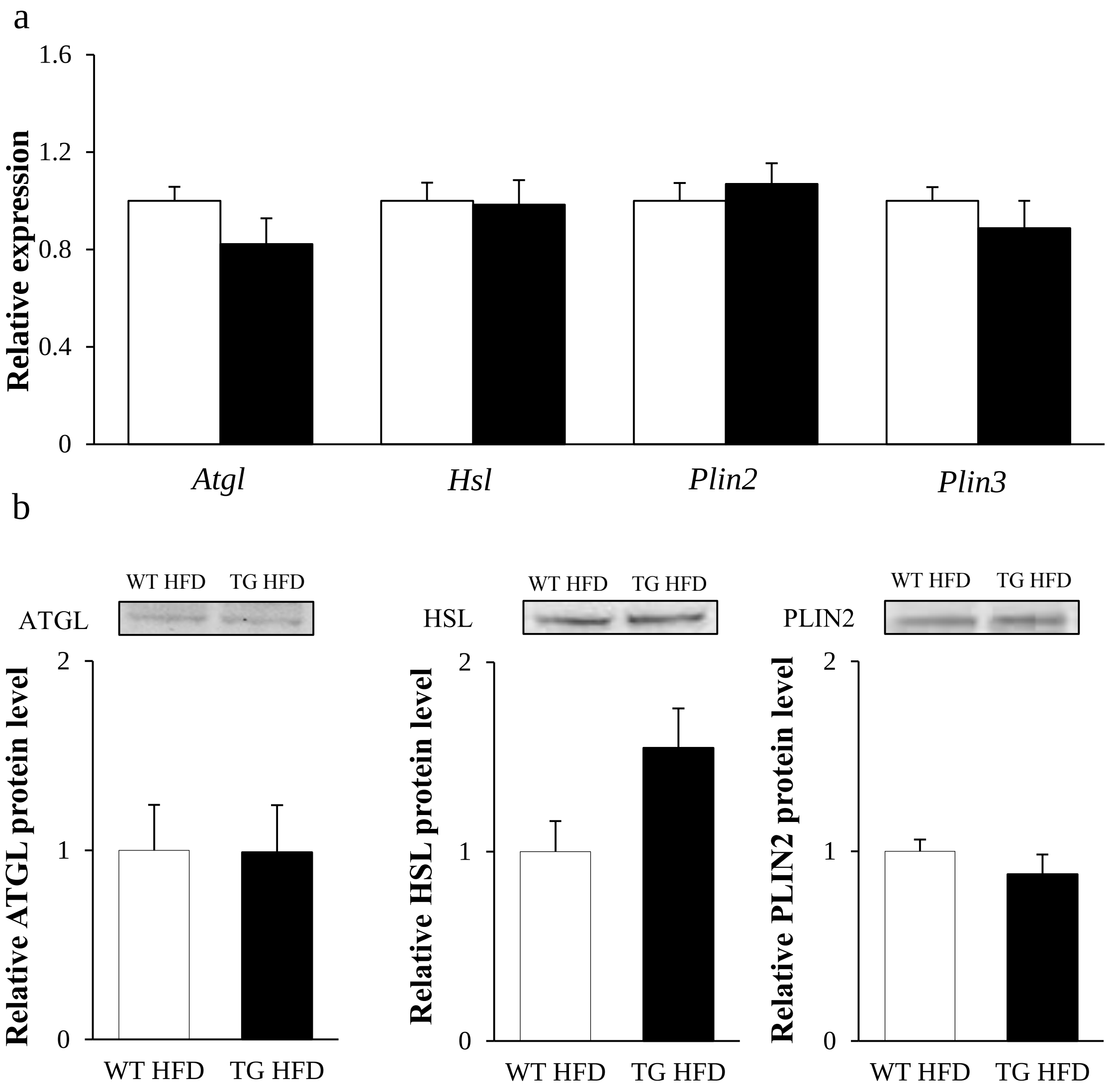


**ESM Figure 9. Measurement of mRNA and protein levels of key lipases and lipid droplet binding proteins in gastrocnemius skeletal muscle of high-fat-fed *Stk25* transgenic and wild-type mice.** (a) Relative mRNA expression of *Atgl*, *Hsl*, *Plin2*, and *Plin3* was assessed by quantitative real-time PCR using commercially available TaqMan Probes (Assay-on-Demand; Applied Biosystems). The expression level of each gene in wild-type mice is set to 1. White bars, high-fat-fed wild-type mice; black bars, high-fat-fed transgenic mice. (b) Protein lysates were analysed by Western blot using antibodies specific for ATGL, HSL, and PLIN2. Protein levels were analysed by densitometry and shown as bar histograms. The level of each protein in wild-type mice is set to 1. Representative Western blot is shown. Data are mean ± SEM from 9-10 mice per genotype. HFD, high-fat diet; TG, transgenic; WT, wild-type.

# ESM Figure 10

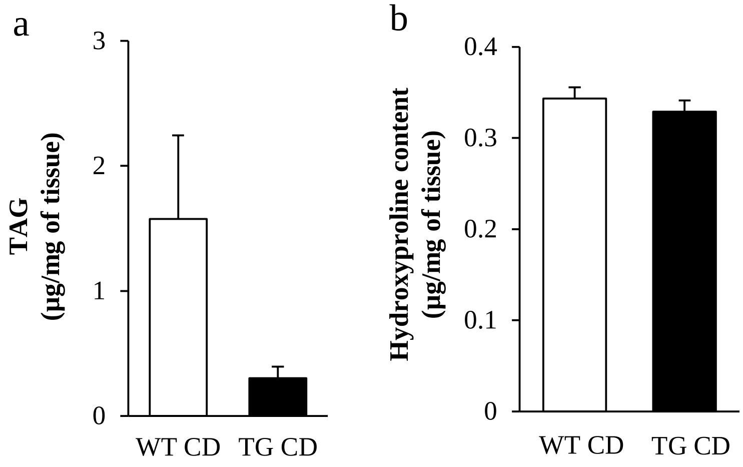


**ESM Figure 10. Assessment of lipid accumulation and fibrosis in gastrocnemius skeletal muscle of chow-fed *Stk25* transgenic and wild-type mice.** (a) Measurement of triacylglycerol levels. (b) Quantification of hydroxyproline content. Data are mean ± SEM from 7-8 mice per genotype. CD, chow diet; TAG, triacylglycerol; TG, transgenic; WT, wild-type.
